# Supplementary material for: Triggers and factors associated with moral distress and moral injury in health and social care workers: A systematic review of qualitative studies
Source: PLoS One. 2024 Jun 27;19(6):e0303013. doi: 10.1371/journal.pone.0303013 (PMC11210881; doi:10.1371/journal.pone.0303013)
Supplement: S2 File — (DOCX) [file pone.0303013.s002.docx]

# Title

Triggers and factors associated with moral distress in health and social care workers: a systematic review of qualitative studies.

# Start Date

14.06.2021

# Completion Date

03.05.2024

# Named Contact

Dr Emily S. Beadle, PGCert, FHEA, CPsychol. Email: [e.beadle@herts.ac.uk](mailto:e.beadle@herts.ac.uk)

# Affiliation

Department of Psychology, Sport and Geography, School of Life and Medical Sciences, University of Hertfordshire, Hatfield, Hertfordshire, UK

# Review Team

Dr Annalisa Casarin, University of Hertfordshire

Dr Daksha Trivedi, University of Hertfordshire

Dr Agnieszka Walecka, Royal Free Hospital

Dr Amy Sangam, Royal Free Hospital

Helen Munro Wilde, University of Hertfordshire

Matthew Winter, University of Hertfordshire

Jessica Moorhouse, Royal Free Hospital

# Funding

None

# Review Question

The aim of the review was to examine the views and experiences of moral injury/distress in healthcare workers employed across a range of clinical settings. Specifically, the primary objective was to explore the causes and triggers of moral injury/distress in Health and Social Care Workers (HSCW) as described in qualitative studies.

In addition secondary objectives were:

• Psychological safety (i.e., feeling able to speak out and/or seek help) and whether this has any impact on mental wellbeing and/or is associated with moral injury/distress in HSCWs.

• Whether diversity/cultural differences were present among HSCW’s influence the experience of moral injury/distress, or consequences.

• The effect (if any) of major events/disasters (e.g., pandemics, low probability/high impact events) on the experience of moral injury/distress of HSCW.

• Insights into preventative treatment strategies and/or interventions as described by HSCWs.

# Searches

Three searches were conducted on 2 main databases: CENTRAL and PubMed (including MEDLINE) between June 2021 and January 2024. Three specialist databases were searched (Scopus, CINAHL and PsycArticles), alongside medRxiv for pre-prints, study registries (e.g., ClinicalTrials.gov, ISRCTN registry), and systematic review reference lists. The “cited by” function in Google Scholar was used for papers selected at full-text screening. In accordance with the protocol, search terms combined relevant key words and MeSH terms related to moral injury, participants and study design. Words were adapted according to the database.

# URL to Search Strategy

<https://www.crd.york.ac.uk/PROSPEROFILES/513196_STRATEGY_20240215.pdf>

# Condition or domain

Moral distress in Health and Social Care Workers (HSCW) describes the psychological unease that is triggered when a professional identifies the ethically or morally correct action to take in a situation but is unable to perform this due to constraints (British Medical Association, 2021). It is a concept first described by Jameton (1984) and further developed conceptually by Morley (2018), where it was also noted that in order for moral distress to occur all that is needed is a moral judgment to have been made and the presence of some external constraint to prevent this (Morley, 2018).

By contrast moral injury has been defined as a range of symptoms (e.g. shame, anger, demoralization) that can occur as a result of an action, inaction or witnessing of actions of others that challenge a deeply held belief or value (Gray et al., 2012; Maguen et al., 2011; Litz et al., 2009; Harris et al., 2015). It has its origins in an military mental health context. Moral injury can occur when the presence of moral distress has a sustained impact on an individual’s physical, mental, or emotional health.

# Participants/Population

## Inclusion:

Healthcare and allied multidisciplinary workers

Including nurses, physicians, consultants, GPs, paramedics/EMTs, occupational therapists, physical therapists, psychotherapists, psychologists, psychiatrists, counsellors, anaesthetists, epidemiologists, nutritionists, patient care teams, audiologists, caregivers, case managers, coroners and medical examiners, optometrists and anyone identified as health personnel, allied health personnel, hospital workers or health and social care workers.

## Exclusion:

Non-healthcare workers (e.g., military personnel, other frontline workers such as police, fire and rescue, hospital management and students of healthcare professions).

Outside of geographical remit stated above.

# Intervention(s)/Exposure(s)

Not applicable - qualitative studies

# Comparator(s)/Control

Not applicable - qualitative studies

# Types of study

## Inclusion:

Qualitative studies (interviews and focus groups, open question survey/questionnaire).

Written in English or Italian

Articles published after 2011, 10 years before the beginning of this review.

## Exclusion:

Review article, book, editorial or similar non-original research study.

Solely quantitative in design or mixed methods.

# Context

## Inclusion:

All healthcare settings including primary and secondary care, public or private practice.

Conducted in North America (Canada & USA), Europe (EU, candidate EU and Schengen Area), New Zealand & Australia.

## Exclusion:

Outside of stated geographical regions.

# Main Outcome(s)

All included studies should have moral injury or distress cited within the primary aims of the study.

# Additional Outcome(s)

Not Applicable

# Data Extraction

## Study selection:

Electronic search results will be downloaded into Rayyan and, where identified, duplicates deleted. Rayyan allows 3 options when marking articles: include, exclude, maybe. The review will follow the PRISMA guidelines. The selection process is:

Abstract/Titles:

- conducted a pilot exercise for inclusion/exclusion using the same 5 abstracts for the entire screening team to calibrate and test the inclusion and exclusion criteria.

- remaining abstracts assessed by 2 reviewers with a third reviewer to resolve any disagreements. This was referred to the whole team where one or more reviewers felt an article had been incorrectly excluded.

Full-text screening:

- conduct a pilot exercise using the same 5-10 full-text articles for the entire screening team to calibrate.

- One reviewer screened the remaining full-text articles, with a second reviewer screening all those marked as exclude.

## Data extraction:

- All reviewers are involved in data extraction. All data extraction will be checked by at least one other reviewer, to ensure consistency.

- the data to be collection included: basic study information (e.g., citation, aims, country), methodological data (e.g., study design and sample), results summary and discussion points (e.g., limitations) to provide context to the reviews and facilitate the findings summary. Any additional sources of results such as supplementary materials are to be extracted in full.

# Risk of Bias (Quality) Assessment

Quality of selected studies will be assessed using the CASP tool for qualitative studies (https://casp-uk.net/casp-tools-checklists/). Reviewers will work in pairs to independently assess and then compare scores across the ten domains. Once computed a quality score will be applied according to how well the study met each criteria; 0 = not met at all, 1 = somewhat achieved, 2 = achieved. The overall score will range from 0 to 20, scores of <10 will be rated 'poor', scores 10-15 'fair', scores 16-18 'good' and scores of 19-20 'excellent'. In order to address any issues, the reviewers met to discuss their decision and to consolidate the final quality appraisal decisions.

# Strategy for Data Synthesis

This review will follow a thematic analysis approach on the verbatim quotes as part of a three-stage process; line by line coding of text, generation of descriptive themes and the development of analytical themes, following guidance from Thomas & Harden (2008), Sandelowski & Leeman (2012) and Shaw et al (2021).

Verbatim findings will be entered into NVIVO and then coded by individual reviewers, line-by-line into codes in order to begin organising the data. (coding is to be annotated with reviewers’ comments but at this point no structuring of codes or further categorisation), coding was undertaken using an inductive approach but with consideration of the research questions.

As multiple people will be coding, codes will undergo continuous review to assess patterns across articles and coders. All reviewers will look for patterns and links between the codes, prior to developing analytical themes. Codes will describe patterns in the data which can then be grouped into categories to develop themes. Using only verbatim quotes, the results will be considered beyond those of the primary studies to develop explanations for the triggers, factors and mitigators of moral distress and injury. Analytical themes are generated by going beyond the summary and conclusions of the original studies. These analytical themes will be considered by a smaller group of review, including a mix of researchers and clinical staff to ensure reality checks on interpretation and to minimise impact of personal bias.

# Analysis of Subgroups or subsets

As this a narrative synthesis, little subgroup analysis can be explored. However should there be sufficient data, the following subgroups may be discussed:

- country, ethnicity, sex, gender and age

- profession (setting; specialisation).

# Types and method of review

Systematic synthesis of qualitative studies

# Health Area

COVID-19, Mental Health

# Language

English

# Country

UK

# Dissemination Plan

Findings to be published in an Open Access peer reviewed journal.

# Keywords

Health Personnel; Qualitative Review, Moral Injury, Moral Distress
