# Supplementary material for: Triggers and factors associated with moral distress and moral injury in health and social care workers: A systematic review of qualitative studies
Source: PLoS One. 2024 Jun 27;19(6):e0303013. doi: 10.1371/journal.pone.0303013 (PMC11210881; doi:10.1371/journal.pone.0303013)
Supplement: S3 File — (DOCX) [file pone.0303013.s003.docx]

| **Author, (Year), Country** | **Aims** | **Sample/ Settings/ Recruitment** | **Age/ Gender/ Ethnicity/ Religion** | **Data collection (qual) methods/ design** | **Theoretical approach/ Analytic process** | **Key themes reported in results** | **Researcher definition of MI/MD** | **Causes of MI/MD** | **Consequences of MI/MD** | **Solutions/ Strategies to MI/MD** | **Participants definitions of MI** | **Limitations identified by authors** | **Implications of the findings by authors** |
| --- | --- | --- | --- | --- | --- | --- | --- | --- | --- | --- | --- | --- | --- |
| Ahokas et al., (2023), Finland | Examine care leaders’ experiences of moral distress in their daily work in older adult care. | 8 Care leaders in older adult care setting. | 28-62yrs, all female, ethnicity and religion not reported | semi-structured interviews online. | Content analysis | Five themes; MD arises from a lack of time. MD contributes to a sense of inadequacy but also a sense of responsibility. MD arises from an imbalance in values, Increased knowledge and open discussion help reduce moral distress. Reflection, increased support and increased resources can reduce moral distress. | Used alongside the terms “ethical problems” and “ethical stress,” moral distress is defined as a psychological imbalance that occurs when people are aware of the ethically appropriate action that a situation would require but cannot perform this action due to external obstacles | Having to make priority decisions between patients, inadequate staffing levels and less qualified staff, and/or less interested in the care of older adults. Economic values superseding or shaping ethical values of organisations, being caught in the middle when family and patient opinions don't align. This study was undertaken during the pandemic and the authors concluded that this caused an increase in MD mainly due to the impact on time and workforce. The need to employ underqualified temporary staff meant competency was sometimes lacking. | Moral distress was seen to contribute to a sense of inadequacy – but also responsibility. The participants experienced that they were unable to maintain a sense of responsibility for patients to the extent they would like | Good relationship with managers. Greater support from senior managers more training and lectures on MD would be useful. On the individual level, time to reflect on current and future work soliloquising (engaging in self-talk) or generally reflecting on the day's events were thinking positively about the future and/or realising that opportunities to influence matters and act differently in the future do exist. taking a moment at the beginning of or during the day to gather and write down one's thoughts and reread these thoughts at the end of the day, that is, therapeutic writing. | N/A | All female participants. The authors suggest the sample size is sufficient. | MD is experienced daily and increased during COVID. Does not really go further in discussing the implications of this research. |
| Arends et al., (2022), Netherl&s | Explore whether nurses in inpatient hospital settings experience MD when involved in life-prolonging treatment in elderly care/advanced disease. | 23 Nurses (14 university hospital, 9 general hospital)/ mix of oncology, haematology, cardiology, geriatric & surgery wards/ Snowball sampling. | 20-30yrs (4), 31-40yrs (2), 41-50yrs (7), 50+ yrs (9)/ 19 female & 4 male. *Ethnicity & religion not reported.* | Semi-structured interviews. Originally face-to-face but changed to telephone due to Covid-19 p&emic. | Thematic analysis with independent double coding. Discussions & collaborative creation of coding tree. | 1. Need to be involved in decisions.  2. Doubting decision for treatment.  3. MD due to treatment.  4. Perceived negative consequences for patient care. | *Morley et al., (2019)* | Decision making processes (feeling exclude, need to be involved), doubt of treatment decisions. | Poor patient care. | *Not reported.* | *Not reported.* | Change of interview setting due to COVID p&emic prohibited non-verbal communication. Focus on nurses. | Nurses need to be given a clear role in decision-making, formal involvement is not always necessary but should be more aware of their role. |
| Beck et al., (2022),  USA | Describe system level factors in US healthcare that contribute to MD among paediatric doctors. | 40 residents & attendings at 4 university children’s hospitals/ purposeful sampling. | Residents mean age 29.4, attendings mean age 41.3/ 8 male 32 female/ 25 Caucasian, 2 black, 7 Asian, 4 Hispanic, 2 other/ *religion not reported.* | Semi-structured telephone interviews. | Inductive approach to thematic analysis with constructivist paradigm. | 1. norms of medical education & practice of medicine. 2. Corporatisation of the US health system.  3. Implementation of protocols & policies 4. Resource constraints & allocation decisions. | "Moral integrity is seriously compromised, either because they feel unable to act in accordance with core values & obligations, or attempted actions fail to achieve the desired outcome". | Environment cultivated for MD by having a norm of action over restraint. Hierarchy also highlighted. Corporatisation of US healthcare system. | *Not reported.* | *Not reported.* | *Not reported.* | Most at rank of assistant professor, only residents & hospitalists. Sample was predominantly white females. | Future focus on determining ways to re-frame the hierarchy among physicians; identify collaborative system-level initiatives to foster the well-being of everyone on the health-care team. |
| Bernhofer & Sorrell, (2015),  USA | Investigate the feelings of MD described by nurses who felt they were constrained in provision of optimal pain management to hospitalised patients. | 48 nurses in a large tertiary care hospitals surgical units/ invitation sampling. | 25-59 yrs of age, 42 females, 4 males/ 43 Caucasian, 5 African American/ *religion not reported.* | In-depth interviews. | Grounded theory. | Feelings about being unable to manage patient's pain & barriers to managing pain. | Jameton (1993) definition "feelings that are painful... disequilibrium that occurs when nurses find themselves in situation where they feel unable to do the right thing". | Being unable to manage a patient’s pain, poor communication with peers, poorly educated staff, suffering caused by knowledge with barriers to practice, lack of reflective practice on emotions. | Long term stress, strained relationships with medical colleagues. | Provide reflection opportunities, improve pain management education, adapting the teamworking dynamic to make nurses less reliant doctors to manage pain relief, institutional change. | *Not reported.* | Single hospital study, secondary analysis not specifically about MD. | Potential to adopt new strategies for nurses to manage pain in the acute care setting. |
| Bourne & Epstein, (2021),  USA | Gain insight into the experiences of primary care providers regarding MD, including causative factors & mitigation strategies. | 12 primary care physicians & nurses in an academic medical centre/ *sampling recruitment not reported.* | Mean age 40.25, 10 females, 2 males/ 9 Caucasian, 2 black, 1 other/ *religion not reported.* | Semi-structured interviews (face-to-face or telephone). | Conventional content analysis. | 1. Policies & procedures that conflict with patient needs.  2. The unpredictable nature of primary care.  3. Need to “bend the rules”.  4. Lack of accountability.  5. Lack of support staff. | MD occurs when healthcare professionals know the ethically appropriate action to take but are constrained from taking action by factors beyond their control. As a result, patient care & the moral integrity of clinicians are compromised (Jameton, 1993). | Internal constraints, named as perceived powerlessness, sense of responsibility, socialization to follow orders, emotional toll of the job, competing obligations, fear of mistakes. | MD must be recognised in order to mitigate consequences. | 1. Promote discussion & debriefing of distressing situations with colleagues. 2. Improve communication at all levels of leadership & clinic hierarchy  3. Improved accountability when policies & procedures were not followed.  4. Improve access to resources. 5. Increase clinic efficiency  6. Increase training for staff  7. More control over own patient care schedules. | *Not reported.* | Small sample size. All participants practiced within the same medical centre, impacts generalisability. Ethnic & gender bias (mainly white & female). Participants self-selected. Interpretation of qual data may also be subjective to researcher bias. | MD is not solely a nursing or acute care phenomenon. |
| Brasi et al., (2021),  Italy | Explore the causes of morally distressing events, feelings, & coping strategies in the nursing population. | 40 (12 dropouts) nurses in a teaching hospital/ snowball sampling. | *Age not reported*/ 23 female, 4 male/ e*thnicity nor religion reported..* | In-depth interviews. | Interpretive phenomenological analysis. | 1. Causes of MD.  2. Feelings & emotions experienced in morally distressing events.  3. Factors that worsen or influence the experience of MD.  4. Strategies for coping with MD.  5. Recovering from morally distressing events.  6. End-of-life care. | MD is a challenge that arises when nurses hold an ethical or moral judgement regarding a specific treatment or when the care that they must provide is in opposition to their personal or professional values. | lack of expression of will, loneliness at the end of life, medical decisions not shared, inability to act, failure to communicate prognosis, not telling patient the whole truth. Intimacy with patients & family members leads to greater emotional involvement. | Afterthoughts, defence mechanisms, changes in thought & practice, drive for continuous improvement. | Dialogue with patient’s families, consultation with medical personnel, enhanced supportive courses for end of life care as current end of life courses do not provide st&ard methods for the management of morally distressing situations. | Nursing staff experience MD when forced to treat a patient in a manner contrary to their own professional & personal values. | Results not replicable because it's qualitative data. Didn't consider gender, religious beliefs, & any previous personal experiences with end of life care. | MI is easier to solve if identified earlier. Moral sensitivity of healthcare workers could be increased through team discussions, using a structured & guided debriefing format ensures that each nurse will compare their own feelings & coping strategies with others. |
| Clark et al., (2022),  USA | Explore how resilience, job satisfaction, & MD effect ED nurse engagement. | 15 ED nurses in multiple hospitals/ purposeful sampling. | 18-30 (1), 31-40 (5), 41-50 (3), 51-60 (2), >60 (4)/ 1 male 14 female/14 white, 1 other/ *Religion not reported.* | Semi-structured interviews. | Theoretical qualitative content analysis. | 1. Resilience & age.  2. Resilience & nursing tenure.  3. Job satisfaction & workplace engagement.  4.MD & workplace engagement. | *Not reported.* | lack of equipment, team collaboration, hostile workplace, poor communication, inability to care for self at work, poor staffing, real or perceived lack of safety, lack of constructive feedback. | Disengagement from work. | Building teamwork, connections with staff, issues being heard by management. | *Not reported.* | *Not reported.* | *Not reported.* |
| Demir et al., (2023), Sweden | To describe nurses’ and specialist nurses’ experiences of moral distress and how it affects daily work in surgical care. | 12 Nurses and specialist nurses in surgical depts in 2 hospitals. | *24-47 (32)/11F& 1M/ ethnicity and religion not reported* | semi-structured interviews in time and place of pt choosing | Framework analysis using Morley (2020) theoretical framework | Findings were not labelled as themes (rather categories) however they were grouped according to sources of moral distress and the three main themes were experiences that lead to moral distress, perceived consequences of moral distress and strategies in case of moral distress. Organizational reasons, staff availability and nurse–patient ratios were presented as reasons for moral distress. Lack of time in terms of a never ending to do list, and the documentation system (time taken and not user friendly) were listed as organisational reasons. Demands from all different directions were also included as a sub-heading. The findings then moved on to discuss consequences relating to nurses (emotional) and patients (lack of resource relating to patient safety). As well as impact for current and future healthcare. Strategies for dealing with moral distress was also discussed in two further sub-themes. | Look to Morely et al (2020) definition of moral distress. Moral distress may be sub-categorized according to the source of distress, for example ‘moral uncertainty distress’. It is of importance to capture the real-life experiences of nurses perspectives of moral distress to obtain all dimensions (Morley et al., 2020). | In the introduction cite other research - often a consequence of staff shortages, inflexible guidelines, a lack of communication, complex documentation or other organizational factors. Some of the causes of moral distress are local, while others are due to the larger healthcare system (Jameton, 2017; McAndrew et al., 2018; Oh & Gastmans, 2015). Cho et al. (2020) note that due to the high workload of today's nurses, priorities are required such that patients' physiological needs are met first. Emotional support is lacking or, unfortunately, must be a lower priority. Understaffing generates time constraints, which in turn have a significant impact and consequences for nursing quality and patient safety (Cho et al., 2020). Reasons or things leading to moral distress were captured as sources demands. | Emotional consequences for nurses (various emotions, such as inadequacy, sadness and frustration. Furthermore, the participants noted that prioritizing questions about who should receive attention first was a daily dilemma), lack of resources (causing need to constantly reprioritise, neglecting holistic care and) and implications for patients in terms of safety. Consequences for current and future healthcare focussed on the likelihood that nurses would leave. | Well functioning teams, nurses as supervisors on the team, good communication, opportunity for reflection. Better conditions - better understanding from senior staff, delegation of some tasks and better salaries. Competency development for nurses was also mentioned briefly. | N/A | recruitment involved participants registering interest in the study. Perhaps an unnecessary barrier to recruitment. Mainly females. Data collection may have been affected using digital interviewing instead of face-to-face interviews, and the interviews were relatively short with an average time of 23 min. | One recommendation regarding the introduction of care weight measurements seems unrelated to the results and is poorly explained. The implications for this research section focus on reducing insecure work conditions to avoid the need to recruit. This doesn't seem to follow from the findings. Support structures to enable nurses to speak up is another "implication for practice" but is not really covered in the findings. |
| Denham et al., (2023), UK | Examine experiences and impact of COVID on moral injury and distress among health care workers. | 20 various roles within NHS in physical and mental health/located in trusts in NW of England/subsample of larger study sample | *27-60 (43)/ 15F&5M/ 17 White British, 1 Asian, 2 other* | Semi-structured interviews | Critical realist perspective using thematic analysis | Three main themes with sub-themes. 1. Attitude towards moral injury 2. experiences of potentially morally injurious events, and 3. consequences of moral injury. Subthemes 1a. Nature of role in shaping attitude towards moral injury 1b. A normal experience. 2a. Unsafe work conditions. 2b. Let down by those in charge. 2c. Disagreeing with instructions/actions from those in charge 2d. Having to make difficult decisions. 2e. Reduced quality of patient care. 3a. Emotional impact of moral injury. 3b. Questioning self, life and purpose. 3c. reduced job satisfaction. | Talks about origins in military context. | COVID specific (first 2) - feeling unsafe at work with a lack of PPE, feeling let down by the government and superiors , disagreeing with actions and instructions of those in charge , having to make difficult decisions and ultimately, providing a reduced quality of patient care that could compromise staff and patient safety | Range of emotional responses which led to some feeling loss of identity and to question self, role and life. Extreme physical and emotional exhaustion. Reduced empathy towards others and poorer patient interactions. Reduced job satisfaction. Consequences were also included in direct relation to COVID - fear around lack of PPE and implication for care worker's families in terms of risk. However, theses consequences are not directly in relation to moral injury so not included here. High turn over rates (this is picked up in the discussion). Cite turnover rates of 10-12% for NHS trusts. | Authors recommend that psychosocial education would help people understand the concept better. Organisations should improve their channels of communication with staff, promote team discussions, acknowledge work-place difficulties and offer explanations where possible for the reasoning behind difficult decision-making. This would facilitate greater trust. | N/A | Findings from a single region of England (but one of the worst affected by COVID). lower representation of community and mental health care workers compared with those in hospital settings. | Participants didn't always ackowledge MI, despite reporting signs and symptoms. This points to the need for psychosocial education. Staff access to psychological support is required. Organisations should improve their channels of communication to promote team discussion and trust. Need development of specific interventions and strategies of support needed for healthcare workers. |
| Deschenes et al., (2023), Canada | aimed to explore what pediatric critical care nurses identify as a needed intervention to minimize moral distress. | 10 pediatric CC nurses/2 pediatric CC units in urban tertiary care hospital/purposive and snowball sampling | 20-29=2, 30-39=8/ all female/ ethnicity and religion not reported | Semi-structured interviews | Unclear but descriptive inductive coding | Lack of communication. Futile Care. Difference of opinions related to patient care. Staff dynamics and unit culture. | "Moral distress “arises when nurses are unable  to act according to their moral judgment. They feel they know the right thing  to do, but system structures or personal limitations make it nearly impossible to  pursue the right course of action” (Canadian Nurses’ Association, 2017)." | Lack of communication. Futile Care. Difference of opinions related to patient care. Staff dynamics and unit culture. | not reported | "(1) “I’m sorry, there’s nothing else”:  increasing supports for patients and their families; (2) “someone will commit suicide”: improving supports for nurses with subthemes (a) receiving help to navigate  their psychological and emotional distress; and (b) strategies to build morale and  enhance unit culture; (3) “everyone needs to be heard”: improving patient care  communication; and (4) “I didn’t see it coming”: providing education to mitigate  moral distress." | N/A | single point of time during pandemic. Rely on self-report. Sample bias due to recruitment. Female only (75% of pop). None who worked >12 years. | explores what nurses would consider suitable intervention and their own needs for these. |
| Fantus et al., (2023), USA | identify MD triggers among a sample of HSWs across the state of Texas during the COVID-19 pandemic | 43 health care social workers/ inpatient, outpatient or hospice/long-term care/Purposive and snowball | 27-62 (38)yrs/ 92% female/ 33% Hispanic | semi-structured online interviews | Vincent’s (2020) model that conceptualises MD as emerging across three levels: (i) patient care decisions; (ii) team/unit-level; and (iii) system level./directed content analysis | (i) Patient care decisions were associated with constraints influencing continuums of care, with sub-themes relating to discharging and end-of-life care. (ii) Personal care decisions were associated with constraints on personal well-being that impeded best patient care, with sub-themes relating to health and safety and professional efficacy. (iii) Unit/Team decisions were based on limits to cohesive team building, with sub-themes relating to interdisciplinary communication and interpersonal hierarchies. (iv) System-level decisions were associated with constraints that reflected broader organisational policies and practices, with sub-themes connecting to workplace culture and visitation policies. (v) Social justice considerations were sources of MD that derived from federal policies and societal contexts outside of the health system, with sub-themes drawing on systems of oppression and politicisation of COVID-19. | MD psychological disequilibrium that arises when institutional constraints obligate an individual to carry out a task that violates their professional and/or personal ethics and values | MD is triggered by: (i) patient care decisions; (ii) personal care decisions; (iii) team/unit decisions; (iv) structural decisions; and (v) social justice decisions. |  | supervisors ought to be trained in identifying and addressing MD in pre-emptive, rather than reactionary, approaches. MD ought to be integrated into social work curricula to prepare students entering the field; continuing education may be an approach to facilitate supervisory skills and competencies to best mitigate long-term stressors | N/A | Analysis relying on participant narratives and limitations may include social desirability bias and recall bias. One state example (Texas) | Health systems need to show investment in HSWs’ health, safety and well-being and consider how to incorporate social justice initiatives into workplace culture |
| Forozeiya et al., (2019), Canada | Describe intensive care nurses’ experiences of coping with MD. | 7 ICU nurses/ purposeful sampling. | *Age not reported*/ All female/ All Caucasian/ *Religion not reported.* | Semi-structured interviews. | Thorne’s approach to interpretive description. | 1. Going against what I think is best.  2. MD – It’s just inherent in our job.  3. It just felt awful.  4. Dealing with it. | ‘‘Perceived violation of one’s core values & duties, concurrent with a feeling of being constrained from taking ethically appropriate action” (Epstein & Hamric, 2009). | Situations where they would have to act in accordance with their beliefs as opposed to their own, questioning the decisions of others & lack of information given to families, circumstances in which patients’ wishes were contested by family members, their perception of how end-of-life care planning was (mis)managed by ICU physicians, nurses’ MD was shaped by the social context of their practice. | Feeling stressed, frustrated & anxious, insomnia & exhaustion, withdrawing from family & friends, & dreading going into work. Many had considered working less hours or contemplated ending their employment in the ICU. | Knowing their patients’ wishes, coping as dynamic processes of both turning away from & turning toward their MD, avoidance/distraction, seek the support of those with a contextual underst&ing of the ICU environment, reading written accounts of other nurses about their morally distressing experiences, able to use their skills, experience, & knowledge to work through the distress & ‘move with it’. | Going against what I think is best, performing an action that was inconsistent with their perception of what was right.”I’ll call it the duality because it’s a conflict inside”. | The experiences that the participants shared were influenced by the context of an academic hospital. All the participants were Caucasian females, therefore the influence of gender &/or culture on the experience of coping with MD could not be deduced. Most of the participants were relatively inexperienced. | Explore interventions that promote social support & involve innovative strategies to enable nurses to have time to attend activities pertinent to patient care, such as family meetings, in an effort to support shared decision making & enhance nurse-physician communication. |
| Foster et al., (2021), Australia | To explore Australian midwives’ experiences & consequences of MD. | 14 midwives in rural tertiary & private practice/ purposeful sampling. | *Age not reported*/ All female/ *Ethnicity not reported*/ *Religion not reported.* | Semi-structured online interviews. | Thematic analysis/ naturalistic enquiry. | 1.Experiencing moral compromise 2.Experiencing moral constraints. 3. Dilemmas & uncertainty. 4. Personal & professional consequences. | Psychological suffering following clinical situations of moral uncertainty &/or constraint, which result in an experience of personal powerlessness where the midwife perceives an inability to  preserve all competing moral responsibilities. | Navigating practice values, termination of pregnancy, cumulative effects. Hospital culture & hierarchy, fear & uncertainty. | Withdrawal from social life (family & friends), lack of trust in own judgement, worse patient care. | *Not reported.* | *Not reported.* | Sample size, generalisability, sample bias. | Prevalence of MD is still unknown but in-depth detail of experiences in midwives. Hierarchical & oppressive services are described in detail. |
| French et al., (2021), UK | Investigate NHS staff experiences of burnout & betrayal-based MI, in which a trusted authority betrays ‘what is right’. | 16 clinical staff in the NHS (nurses, doctors, OT’s, trainee clinical psychologists, paramedics)/ convenience sampling. | *Age not reported*/ 4 male, 12 female/ 15 white, 1 Asian/ *Religion not reported.* | In-depth interviews. | Critical realist approach. | 1. Ab&onment as betrayal.  2. Dishonesty & lack of accountability.  3. Fractured relationship with management of the NHS. | The distress that can arise from actions or lack of action that violate one's moral or ethical code (Litz et al, 2009). | Violation of trust by individuals or the organisation, leadership behaviour - lack of accountability. | Breakdown of relationship between staff & management in relation to NHS engagement. | Opportunities for other types of systemic & leadership-based interventions which may complement existing guidance & support, to manage feelings of betrayal & loss of trust. | Places MI & burnout in the context of betrayal & loss of trust in leadership. | Small sample size, use of qualitative instead of quantitative data, limited to small sample sizes of specific roles within organisations, lack of ethnic/cultural diversity. | In the case of MI due to betrayal, further strategies need to be developed by organisations to improve management of individuals to prevent this from happening. |
| Gagnon & Kunyk, (2021), Canada | Underst& the MD experiences of paediatric nurses in end-of-life care for children. | 7 paediatric intensive care nurses across 6 paediatric ICU’s/ invitational sampling. | *None reported.* | Unstructured interviews. | Narrative enquiry. | 1. Dignified death & balancing best interests.  2. Burden of insider knowledge. 3. Environmental constraints on nursing roles & responsibilities. | It is a relational concept characterised by a personal experience of serious moral compromise, not an inadequacy of the individual, but rather originating within the broader systemic organisational contexts & practices. | *Not reported.* | *Not reported.* | *Not reported.* | *Not reported.* | *Not reported.* | *Not reported.* |
| Gherman et al., (2022), Romania | Explore the influence of healthcare ageism on nurse MD. | 25 nurses across specialities (pulmonology – 2, OB – 4, neuro – 1, pallative – 3, surgical – 5, ICU – 2, oncology – 1, radiology – 1, nuclear med – 1, nursing home - 3/ snowball sampling. | 26-55yrs/ 2 male, 23 female/ *Ethnicity not reported*/ *Religion not reported.* | Episodic semi-structured interviews. | Constructivist thematic analysis. | 1. Macro-level triggers.  2. Meso-level triggers.  3. Micro-level triggers.  4. Emotional experiences of MD. | McCarthy & Monteverde (2018). | Macro-level (scarcity of resources ageist or self-ageist mentalities, systemic corruption, insufficient training, ageist laws, policy & ageist COVID-19 restrictions). Meso-level (discrimination, stereotyping, prejudice, 'hush-hush' culture, (in)subordination to ageist norms, peer conflict). Micro (stereotyping, prejudice, discrimination). | Apprehensive of speaking out.  helpless/powerless, traumatized, overly emotional, detachment. | *Not reported.* | *Not reported.* | Homogenous national identities of sample. Data from patients & families needed to corroborate findings. | Concept of MD gains comprehensive power if articulated with the dimensions of ageism. |
| Hancock et al., (2020), Canada | Explore personal & organisational factors that contribute to burnout & MD in an academic ICU team. | 35 (21 ICU nurses, 8 respiratory therapists, 6 physicians) in an academic ICU/ convenience sampling. | *Age not reported*/ 30 females, 5 males/ *Ethnicity not reported*/ *Religion not reported.* | Focus groups. | Thematic analysis. | 1. Organizational issues. 2. Exposure to high intensity situations  3. Poor team experiences. | MD is believed to occur when individuals are placed in situations that are at odds with their core values & beliefs & that they have little power to change. | A lack of adequate physical resources, lack of adequate staffing, ineffective organisational policies, & disengaged administration. Decisions made by the health organization at the expense of quality patient care or the physical &/or mental health of its employees. . .provision of futile care & end of life care, advocating for appropriate patient care, the healthcare provider patient/family relationship, & workplace violence. lack of control in the workplace, lack of appreciation by team members & administration, fragmented patient care, & negative team dynamics. | Physical & emotional toll, impact on home life, a decrease in compassionate patient care, strong & negative emotional reactions. | Organisational; improve staffing, invest in education, improve infrastructure, regular debriefing, de-stigmatise the need for support, address workplace violence. | *Not reported.* | There may have been confirmation bias in the focus group members, which may have led to an overestimation of the problem. May not represent the opinion of the majority of the ICU team as the focus group enrolment was self-selected. Differences between the groups may not reflect true differences, but may reflect the organic nature of focus group conversations. | There were worthy suggestions identified by participants for building resilience. |
| Helmers et al., (2020), Canada | Explore how paediatric CC healthcare providers respond to MD, to inform the development of interventions & resources that support a healthy moral practice. | 17 (7 in focus group, 10 interviewed) paediatric ICU nurses in a tertiary paediatric hospitals CC unit/ convenience sampling. | 30-60yrs/ *Gender not reported/ Ethnicity not reported/ Religion not reported.* | Focus groups & semi-structured interviews. | Sequential two-phase iterative approach. | 1. Factors influencing distress.  2. The evolution of practice over time & its impact on MD.  3. Strategies participants have developed to address their distress. | “The experience of knowing the right thing to do while being in a situation in which it is nearly impossible to do it.” (Jameton). | Social factors included a perceived lack empathy, the perception that efficient care delivery was prioritised over the needs of individuals, & the perception of a culture of stoicism. Environmental factors included scheduling constraints (i.e. self-scheduling) negatively impacting a sense of team & social networks, & a paucity of resources. | *Not reported.* | Active (personal actions), reflective (perspective-making), & external (creation of formal support). | *Not reported.* | Generalisation was neither sought nor achievable. As such, no broad inferences can be drawn in relation to the nature of MD from this small sample. | Deeper underst&ing of how experienced practitioners in the ICU manage & mitigate MD - example of how a clinical unit can identify & cultivate methods for building moral resilience. |
| Henrich et al., (2016), Canada | Examine the causes of MD in diverse ICU units within the community & tertiary ICU’s. | 56 mixed (19 nurses, 4 nurse leaders, 13 physicians, 20 other) from 13 ICU’s in Vancouver/ convenience sampling. | *Not reported.* | Focus groups & telephone interviews. | *Not reported.* | 1. Quality of care.  2. Amount of care provided.  3. Inconsistent care plans.  4. End-of-life decision making.  5. Poor communication.  6. Interactions/conflict between ICU staff & family.  7. Recommendations for patient care ignored by other staff.  8. Lack of support or resources. | MD is the anger, frustration, guilt, & powerlessness that health care professionals experience when they are unable to practice according to their ethical st&ard. Any negative emotions that the person experienced in response to a conflict between the care they think should be provided & the care that is provided. | Concerns about other providers' care. Lack of end-of-life conversation. Balance in amount of care provided. Inconsistent care plans, poor communication, end-of-life decision making. Conflict between ICU staff & families. Recommendations of ICU staff for patient care ignored by other ICU staff. Lack of resources, lack of support from management. | *Not reported.* | When decisions are made to switch to comfort care, implement this change as quickly as possible & do not delay until the next attending takes over unless there is a compelling medical reason. Provide training to physicians on how to provide end-of-life news to families in a Adopt a clear & direct way to avoid misunderst&ing or uncertainty by families on the patient's condition. Clarify with all staff the meaning of how individual care should & should not be provided. Training physicians on how to present end-of-life plans to families in a way that meets the needs of individual families. Whenever possible, include nurses, social workers, & patients in family meetings about end-of-life care. Begin changing the culture around asking for help. | *Not reported.* | The use of focus groups provides insights into participants' experiences & perspectives, but the findings are not generalisable. Low participation rate by profession, except for physicians, may limit the generalisability of these findings. | Interventions to address MD should consider the specific causes, the target groups & settings where they apply. |
| Howard et al., (2023), USA | "The purpose  of this study was to explore the experience of moral distress in  occupational therapists during the time of COVID-19" | 18 Occupational therapists/various settings/ stratified purposive sampling | *25-34 (7), 35-44 (8), 45-54 (1), 55+yrs (2)/ 1M 17F/ 16 white, 1 Asian, 1 Black* | semi-structured interviews/ hermeneutical phenomenology | hermeneutical phenomenology | experiences, effects, managing moral distress |  | change in role, PPE, uncharted waters (untrained experiences), PPE patient challenges and violating patient autonomy. | burnout, employment complications, impact on life roles. | professional help, talk with family or coworkers, used exercise to manage stress. | *N/A* | limited sample, diversity, self-selection bias, predisposed to assume all OT had impacted by MD. | uniquely considers role of OT and the impact of COVID on these professionals |
| Jansen et al., (2020), Norway | Explore the sources & features of MD experienced by acute psychiatric care nurses. | 16 registered psychiatric nurses in 2 mental health hospitals/ purposeful sampling. | *Age not reported*/ 3 male, 13 female/ *Ethnicity not reported/ Religion not reported.* | In-depth interviews. | Thematic analysis. | 1. Experienced dilemmas between nurses' perceptions of capacity & patients' needs.  2. Risk of violence & dilemmas concerning coercion. 3. Experienced physical & mental reactions to MD. | 'MD causes bad conscience & feelings of guilt, frustration, anger, sadness, inadequacy, mental tiredness, emotional numbness & being fragmented. Others feel emotionally flat, cold & empty, & develop high blood pressure & problems sleeping. | Coercive care (only following orders), forced to participate in care/medication decisions that they do not agree with, violent behaviour, insufficient resources, little influence on decision making framework. | Feelings of inadequacy, fatigue, become distant, increase in potential harm for patients, self-doubt, loss of confidence, frustration, anger, sadness,, frustration, loss of meaning, sleep issues, high blood pressure. | Quitting job, taking a year off. An increase in trained staff, better resources. | *Not reported.* | Only 3 interviewees were male out of 16. Participation was voluntary so views may not be representative of wider population. | Exposure to the effect of violence on healthcare staff, importance of further research in acute psychiatric context, further research on gender differences in psychiatric nurses is needed. |
| Jansen et al., (2022a), Norway | Explore how nurses attempt to cope when suffering with MD. | 30 (16 individual interviews & 14 in focus groups) psychiatric nurses in 2 psychiatric hospitals./ purposeful sampling. | *None reported.* | In-depth interviews (16) & focus groups (14). | Thematic analysis. | 1. Sorting thoughts & feelings.  2. Not taking their work home.  3. Loyalty vs. speaking up. | “An unpleasant feeling or a psychological imbalance which arises when one knows what the ethical right action in a certain situation is, but internal or external restraining factors maki it not possible to act accordingly”. Jameton (1984). | *Not reported* | *Not reported* | Managing emotions, not taking work home with them. Loyalty & part of the machine or speaking up. | *Not reported.* | voluntary sampling, localised sample. | Some 'solutions' may lead to greater MD or have other consequences. |
| Jansen et al., (2022b), Norway | Investigate the interaction between political & therapeutic ideals interact with new legislation. How these present conflicting interests in nurses in psychiatric care, causing MD. | 44 (30 interviews & 14 in focus groups) psychiatric nurses in 2 psychiatric hospitals/ purposeful sampling. | *None reported.* | 3 Focus groups (14) & interviews (30). | Thematic analysis. | 1. Challenging behaviour & risk of violence.  2. Minimising the use of restraints & coercion created uncertainty.  3. Ideals & legal changes may frustrate treatment.  4. Consequences for the nursing staff. | “An unpleasant feeling or a psychological imbalance which arises when one knows what the ethical right action in a certain situation is, but internal or external restraining factors maki it not possible to act accordingly”. Jameton (1984). | Moral doubt, restraining & coercive behaviours. | Guilt, bad conscience, sadness, powerlessness, emotional numbness, shame, cynicism, despondency, anger, self-criticism, resignation, withdrawal emotionally from patients, social disconnection, headaches, sleeplessness, weight change & palpitations. High staff turnover, burnout & poor patient outcomes. | Reduce use of coercive treatment, reduce use of unqualified staff, improve available resources. | *Not reported.* | Participation was voluntary so views may not be representative of all nurses. Study is limited in sample size & is local. | Findings highlight the following:  1. Leadership needs to be willing to listen to & act upon nurses ideas & concerns.  2. Leadership should actively encourage an ethical work climate.  3. Equip wards with adequate resources & staff  4.Develop clearer guidelines for coercive treatment. |
| Keilman et al., (2023), USA | This study sought to determine if nurses do care for patients who forgo treatment due to cost (PFTDC) and if so, does this result in an experience of moral distress (MD). | 20 nurses from midwestern community hospital, long-term care facilities and primary care/ convenience sampling | *26-67 (47) yrs/ 1M rest F/ ethnicity and religion not reported* | semi-structured interviews | not reported/ detailed description of steps but exact method type not reported | narratives of PFTDC, strategies to help PFTDC, and the broken US health care system. | Jameton (1984) | patients who cannot be treated due to costs of healthcare. End up in ER because cannot afford non-emergency care. Worse outcomes and multimorbidity problems as a result of not having early/non-emergent care. work the system for patient benefit - cost of time and effort for a temporary fix. Broken system. |  |  |  | *generalisability, unfamiliarity of pts with MD prior to interview.* | end point of this project is to increase nurse advocacy for reforming the US health care system toward greater equity by raising awareness of MD over the perceived inequities. |
| Koonce et al., (2023), USA | gain understanding of nurse meaning-making of morally distressing situations, with particular attention to ethical norms, moral agency and resiliency, and nurse religious/spiritual orientation. | 9 pulmonary care nurses in tertiar care teaching hospital/ purposive sampling | *not reported/ all female/ ethnicity not reported 7 spiritual but not religious, 1 catholic, 1 agnostic, 2 not spiritual or religious* | Semi-structured interview | descriptive penomenological philosophy | meaning-making, rooted in the identity of the “good nurse”: Being true to one’s own values, pursuing ideal patient care (“doing good”), and conforming to/challenging values of the system and culture. Tensions were found between (a) nurse’s own values (b) duty to institutional norms and duty to nurse’s personal code of ethics, and (c) perceptions of institutional support in response to nurse moral distress. Religion was described as a remote source of nurse moral values, among other sources. Spiritual practices were not experienced as sufficient in coping with moral distress at the bedside. | Morley et al (2020) | nurse culture exacerbates moral distress | QoL of patients, patient advocate, prolonged perceived patient suffering | institutional support |  | *Though the interviews provided rich description, the sample was drawn from nurses on the pulmonary unit in one hospital. All participants were female, a gender imbalance that could affect results. Participants identified themselves as not strongly religious, thus only focusing on spiritual or non-spiritual aspects of their experience. Furthermore, interpretations of significant statements, while shared with and validated by the participants themselves, could contain bias not evident to the researchers.* | uniquely considers role of spirtuality/religion in a qual reported study. |
| Kreh et al., (2021), Italy & Austria | Investigate the nature of resilience & stress experience of healthcare workers during the COVID-19 p&emic. | 13 experienced doctors, nurses & psychologists/ invitation sampling. | *Age not reported*/ 2 male, 6 female (interviews), 5 mixed m/f (focus group)/ *Ethnicity not reported/ Religion not reported.* | Interviews (9) & focus group (4). | Grounded theory. | 1. Fear, guilt feelings, frustration, loss of trust & exhaustion.  2.Causal factors: rapidly evolving situation with high uncertainty.  3. Stressors.  4. Resilience factors. | MI is present when there has been (a) a betrayal of what’s right; (b) by a person in legitimate authority (e.g., a leader) &, (c) in a high stakes situation. | MI can be conceptualised in triage, shortage of PPE & non-pharmaceutical interventions. | Psychological distress. | Receiving fast & efficient communication, training & support in PPE use, psychosocial interventions, building resilience, resistance & recovery skills. | *Not reported.* | *Not reported.* | “1. Gathering of first-line experiences & impressions.  2. Steering the steps of a future lesson learning process.  3. Precociously identifying issues that due to the long timeline of the COVID-19 crisis might be of help to countries whose response will be postponed in as well as to all those who are faced with further waves of the p&emic" |
| Lamiani et al., (2021), Italy | Explore physicians’ experiences of MD during the COVID-19 p&emic. | 18 ED & ICU physician (5 ED & 10 ICU, 3 dropouts)in 6 hospitals/ snowball sampling. | Mean age = 46 (SD = 5.24)/ 60% female, 40% male/ *Ethnicity not reported/ Religion not reported.* | Semi-structured in-depth interviews. | Grounded theory. | 1.Being a good doctor in the face of the p&emic.  2. P&emic stressors.  3. Emotions of MD. 4. Individual responses 5. Working environment 6. Moral outcomes. | (Jameton) “The painful feeling that occurs when “one knows the right thing to do, but institutional constraints make it nearly impossible to pursue the right course of action” | Limited healthcare resources. Intensified patient triage. Volume of consequential decisions to make. Changeable selection criteria. Limited therapeutic/clinical knowledge. Patient Isolation. | Moral residue, disengagement, moral integrity. | Avoidance, Acquiescence, Resistance, Reinterpretation. | *Not reported.* | Small convenience sample, results are not generalisable. | The results suggest that in order for physicians to successfully manage MD during the p&emic, it is important to find new ways of enacting their own values by reframing morally distressing situations. In order to ensure a healthy workforce, & prevent job resignation, it is be important to promote a healthy interpretation of morally distressing situations through ethical consultations & psychological interventions. |
| Liberati et al., (2021), UK | Characterise the experiences of those working in English NHS secondary mental health services during the first wave of the COVID-19 p&emic. | 35 (17 psychiatrists, 10 MH nurses, 5 psychotherapists, & 3 clinical psychologists) in community MH teams (EIP, crisis management, acute MH hospital wards, secure forensic services)/  Purposeful & snowball sampling. | *Age not reported*/ 11 male, 19 female, 5 not provided/ 24 white 3 asian, 2 mixed ethinicity, 1 any other, 5 not provided/ *Religion not reported.* | Semi-structured in-depth interviews. | Constant comparative method. | 1. Changes in services & the nature of work.  2. Impacts of Covid-19 related service changes on staff.  3. Wellbeing & the use of support. | The concept of occupational MD & MI was developed in the context of active military combat & emergency response services. It describes suffering felt when staff are involved in, witness, or fail to prevent acts that may not be of any personal threat but transgress deeply held moral expectations. | Greater responsibility, longer shifts to cover sickness, increase in infection control restrictions, loss of opportunity of face-to-face learning, having to make clinical decisions based on second-h& information & reports, loss of therapeutic functions, worry about patients whom were not getting the care they needed, redeployment to assist other services at short notice. | Take personal leave, transfer to another service, use of numerous support services on offer. | Use support services, become mindful of the widespread effect of the p&emic, increase in public appreciation & 'heroes' mindset provided some comfort. | *Not reported.* | No formal measurement or assessment was conducted on the mental health of participants & relied on their own narratives only. Unable to distinguish between experiences of staff from different social groups or investigate the effects of race. Study was conducted over a set period of the p&emic & may not reflect a full experience of MI. | Study is important in demonstrating the importance of informal knowledge sharing & mutual monitoring, known to be essential in maintaining safety in healthcare teams. Schwartz rounds, multidisciplinary forums where staff convene to discuss work, are rich in potential & their role, even in online methods should be further evaluated. |
| Lovato & Cunico, (2012), Italy | Describe nursing practice situations that cause MD as a result of recognition of the ethically appropriate actions, in combination with the impossibility to pursue it. Describe how nurses manage MD situations, & the strategies to cope with them. | 40 nurses in a teaching hospital/ purposeful sampling. | *Age not reported/* 7 male, 33 female/ All Caucasian/ *Religion not reported.* | Focus groups & diaries. | Thematic analysis. | 1. Clinical decisions.  2. Nursing competency.  3. Nurse/ physician collaboration 4. Organisation of care.  5. Safe care. | MD in nursing practice is described as a suffering situation that arises when the nurse is unable to act her/his ethical choices, when institutional constraints interfere with acting in the way she/he believes to be right. | The difference between nursing & medical perspective e.g., pain management, end of life care, mental capacity of patients, value of clinical judgment, support for family, inter-professional respect & knowledge of competency, lack of care time & quality of care. | Moral residue. | Multi-professional meetings, peer discussion of events. | *Not reported.* | *Not reported.* | The need for inter-personal training & development. |
| Matthews & Williamson, (2016), UK | Extend underst&ing of how healthcare assistants construct & manage dem&ing situations in a secure mental health setting. Explore the effect of this environment on healthcare assistants health & well-being, & provide recommendations for effective coping strategies. | 10 healthcare assistants in 2 secure female adolescent wards in a secure MH hospital/ convenience sampling. | 21-43 yrs of age/ 3 male, 7 female/ *Ethnicity not reported/ Religion not reported.* | Diaries & semi-structured interviews. | Interpretive Phenomenological Analysis. | 1. Normalising an abnormal environment. 2. Between compassion & control. 3. Imbalance of occupational dem& & support. | The stress experienced when one is constrained from acting in accordance with one's own set of moral values as a result of external constraints. MD is thought to comprise of emotional responses including feelings of anger, frustration & guilt, feelings which left unresolved may have a negative & enduring consequences on both healthcare professionals & patient outcomes | Feeling that employer has systematically failed to nurture well-being & no sense of healthcare workers psychological needs, feeling constrained by seniors decisions, difficulty of decision making in high-stress environment, organisation promotes depersonalised & detached care, inability to normalise destructive behaviour & self-harm. | Loss of psychological well-being, loss of emotional response to negative stimuli, alienation from profession, struggle to interact with patients, developing humour as a coping mechanism, lack of optimal recruitment & staff retention . | Humour/laughter, use of diaries to aid coping with incidents, increase in organisational support available, & debriefs following incidents. | *Not reported.* | The study used permanent & casual employees whom had worked for at least six months, permanent staff were significantly more likely to suffer from burnout & MD. Majority of participants were degree educated & were working as a healthcare assistant to further their careers. Seven participants were in their 20's & may not have developed skills to manage MD appropriately. | A significant increase in accessible & consistent peer & psychological support may alleviate MD & regain autonomy. Through recognition of clinical issues which cause significant MD to healthcare assistants. Organisations may change the way they provide support resulting in optimal recruitment & staff retention. Keeping diaries are an effective tool in reducing emotional distress & give staff an opportunity to underst& their feelings & reflect on their actions. |
| McCracken et al., (2021), USA | Describe MD as it is experienced by oncology teams in practice. | 32 mixed oncology teams (19 nurses, 4 nursing assistants, 3 physios, 2 chaplains, 1 doctor, 1 case manager, 1 child specialist, 1 occupational therapist) from a medical college & hospital/ convenience sampling. | Age range 23-60 yrs (50% between 23-40 yrs)/ 5 male, 27 female/ 27 white, 2 African American, 2 Hispanic, 1 mixed-race/ *Religion not reported.* | Semi-structured focus group. | Content analysis. | 1. The meaning of oncology care.  2. The rippling effects of cancer.  3. Decision-making barriers are central to the MD experience.  4. Other antecedents of HCPs’ MD. 5. Consequences of HCPs’ MD.  6. Ways to mitigate MD. 7. Burnout in oncology. | MD is a complex issue in health care & presents challenges for healthcare professionals, healthcare organisations, &, importantly, patients & families (Mc&rew et al., 2018). | Frequent exposure to end-of-life care, challenges with pain control & conflicts about goals of care. Feelings of powerlessness & difficulty sharing moral perspective in clinical practice. | Inability to provide patient & family centred care. Loss of compassion. Fatigue & burnout. The author also sites other research - staff turnover & compromised care quality (Lamiani et al., 2017; Mc&rew et al., 2018). | Mitigation strategies - teamwork, early goals-of-care discussions, accessible organisational & unit-based resources. Finding meaning in one's work may protect against burnout. | *Not reported.* | Limited generalisability as study took place in one healthcare site & most participants were nurses. | Practice recommendations included enhanced teamwork, early palliative care involvement, a communication liaison role, & accessibility to mental HCPs. Healthcare team perspectives on MD can guide the development of targeted strategies to mitigate MD. |
| Molinaro, Polzer et al (2023), Canada | to elucidate how the nurses’ counter-stories: (i) re-locate the sources of their moral distress within institutional constraints that fracture their moral identities and moral relationships, and (ii) dis-locate dominant narratives of technological cure by ascribing value and meaning to the relational care through which they sustain moral responsibilities with patients and their families. | 9 pediatric oncology nurses in various settings/ purposive sampling | not reported |  | Theoretical framework of Peter & Liaschenko (2013)./ Lieblich et al (1998) and Laliberte et al (2017) approach | 1- Institutional constraints: idealized moral identities and broken moral relationships. 2- Revaluing relational care: assigning meaning to moral responsibilities. 3- | Peter and Liaschenko’s (2013) theorization of moral distress, which is grounded in the feminist commitment “to changing uneven distributions of power and privilege in everyday life”. "Canadian Nurses’ Association (CNA) Code of Ethics (2002), MD occurs in: [S]ituations in which nurses cannot fulfil their ethical obligations  and commitments (i.e., their moral agency), or they fail to pursue what they believe to be the right course of action, or fail to live up to  their own expectations of ethical practice, for one or more of the following reasons: error in judgment, insufficient personal resolve or  other circumstances truly beyond their control (Webster and Baylis, 2000). They may feel guilt, concern or distaste as a result." | "chronic shortages and faults in the health care system and unresponsive management, which is compounded by  the acute traumas of the specific stresses associated with pediatric oncology nursing... nurses’ moral identities were fractured as a  result of workload demands and the difficulties of having to attend to an expanding range of caregiving responsibilities... influenced by ruptures in moral relationships." |  | *"changes in policy and*  *practice to make nurses, and the full scope of their caregiving, valued."* |  |  | Future research on managerial responses to moral distress would help to elucidate the institutional dynamics that constrain and shape nurses’ moral identities, relationships, and responsibilities and foster more supportive caregiving environments. |
| Molinaro, Shen et al (2023), Canada | To explore stories of moral distress shared by family physicians caring for patients experiencing health needs related to social inequities. | 20 GPs from various primary care, outpatient and community settings / purposive sampling | 25-63 yrs 7M 13 F/ ethnicity and religion not reported | semi-structured narrative interviews | critical narrative approach. | Key themes not clearly reported, but broadly the authors discuss emotional response to moral injury as feeling powerless, helpless, hopeless, frustrated and demoralised. Causes were described as lack of resource and scope of practice forcing them to compromise, and behaviour of patients experiencing broader systemtic constraints. Moral distress rooted in system problems. | Moral injury is regarded as a deep violation of what one believes is right, and can be the identity- changing result of experiencing multiple morally distressing events (authors cite Shay 2014) | Being unable to address the root causes of insufficient resourcing for patients experiencing inequity. GPs are having to work within constraints of a broader system. Conflict, and constraint in being able to provide care. Other causes of ill health that GP not responsible for and can't address. | *The authors suggest that the increase in clinical care responsibility and increase in complex cases is causing GPs to leave. They cite other research that suggests this but don't really draw concrete conclusions from their own findings on this.* | Capitation or salaried models, may offer physicians more time and ability to care holistically for patients with complex needs. However this is not explicitly offered as a solution to addressing moral injury and the authors recognise this would do little to address the upstream deficiencies in the welfare system. Access for patients to MDT such as social workers and legal advisors may help address patient needs better (but again doesn't explicitly address moral injury in GPs). | *Participants described moral distress as a ubiquitous feature of their professional lives; their narratives suggested they were often constrained in their ability to decide on and do what they felt was right for patients.* | Findings may be less relevant in areas/countries with stronger social welfare policy and social safety nets. | The authors concluded that to date, there is limited research on moral injury in primary care, particularly in relation looking after patients experiencing inequity. Participants’ moral distress was associated with remuneration structures and workload demands, and was exacerbated by a lack of social resources, such as housing, and insufficient comprehensive mental health and addictions services, which resulted in strict limitations on their ability to provide comprehensive care. In the discussion talks about how non-compliance may be the result of system limitations influencing patient behaviour. The concluding remarks focuss on the need to change GP renumeration. |
| Morley et al., (2020), UK | Develop a theoretically robust conceptualisation of MD that is meaningful in nursing context, using theoretical & empirical methods. | 21 critical care nurses in trauma (15) & specialist (6) ICU’'s/ purposeful sampling. | 25-54 yrs of age/ 3 male, 18 female/ *Ethnicity not reported/ Religion not reported.* | Phenomenological interviews. | Feminist interpretive phenomenology. | 1. Moral constraint.  2. Moral tension.  3. Moral conflict.  4. Moral dilemma.  5. Moral uncertainty. | MD: one knows the right thing to do, but institutional constraints make it nearly impossible to pursue the right course of action - feelings of anger, frustration, guilt, loss of self-worth, sorrow, anxiety, misery, dread, anguish, depression & nightmare. | Moral events (constraint, tension, conflict, dilemma, & uncertainty, inability to make a moral judgement. | *Not reported.* | *Not reported.* | *Not reported.* | Sample may affect the transferability of the findings. All participants were European & only one participant was from a minority background. The findings presented are based on our interpretation of participants’ reported experiences. | The key to underst&ing & addressing MD - subcategorise distress to ‘moral-constraint distress’ & ‘moral-conflict distress’, to which we would suggest adding ‘moral-uncertainty distress’, ‘moral-dilemma distress’ & ‘moral-tension distress’. |
| Morley et al., (2022), UK | *As Morley et al., (2020).* | *As Morley et al., (2020).* | *As Morley et al., (2020).* | Phenomenological interviews | Feminist interpretive phenomenology | *As Morley et al., (2020).* | *As Morley et al., (2020).* | *As Morley et al., (2020).* | *As Morley et al., (2020).* | *As Morley et al., (2020).* | *As Morley et al., (2020).* | *As Morley et al., (2020).* | *As Morley et al., (2020).* |
| Musto & Schreiber, (2012), Canada | Develop a substantial theory of the process’s nurses use when they experience MD.  1. What situations create MD for mental health nurses in adolescent care?  2. How do mental health nurses experience MD?  3. What do nurses do to ameliorate the experience of MD?  4. What is perceived to be supports & barriers to resolve the experience of MD? | 12 registered nurses in inpatient or community services for adolescents with MH issues/ *Recruitment not reported.* | 26-54 yrs of age/ 4 male, 8 female/ *Ethnicity not reported/ Religion not reported.* | Semi-structured in-depth interviews. | Glaserian grounded theory. | ‘Doing the best I can do' - basic social process participants used when they had an experience that produced MD. The process for this begins with;  1. 'Engaging with dialogue'.  2. 'Experiencing the dialogue'. 3. 'Shifting perspective'. | A situation in which a nurse makes a moral judgement but is unable to act on that judgement due to external constraint. | Inconsistent systems of treatment, contradictory treatment of patients, feeling unheard when trying to address feeling distress, lack of ethical supervision, lack of validation, inability to share concerns, intimidation from senior staff members. | Loss of confidence in ability, talking of leaving, leaving employment, high turnover of trained staff. | Engagement & experience of positive dialogue in a safe space allows for a shifting perspective. Starts with a feeling on possible inadequacy but shifting perspective allows fresh context & validation of participants ability. | Most participants identified an experience of MD following on from an incident involving patient safety, with a feeling of inability to maintain patient safety. | Findings not generalisable due to small study size, narrow focus on adolescent mental health nurse population. | Importance of dialogue in working with ethical issues & importance of clear policies to guide professional practice. Implications for job satisfaction, staff turnover & safer patient outcomes. |
| Pye, (2013), UK | Explore perceptions of doctors & nurses working in an English regional paediatric oncology unit on lived experiences & feelings related to MD, with a focus on team dynamics. | 8 (4 doctors, 4 nurses) in a children’s hospital/ self-selected sample. | *None reported.* | Open-ended semi-structured discussion from hypothetical scenario. | Colaizzi’s (1978) descriptive framework & Riley’s (1996) method of qualitative analysis. | 1. Decision making,  2. Conflict over right to treatment  3. Withholding by participants, the importance of communication with the team. | MD is defined as ‘‘the painful feelings caused by specific situations where the practitioner is convinced of the morally appropriate course of action but is prohibited from carrying this out,’’ (Godfrey & Smith, 2002). | Influenced by participant’s own individual historic experiences. Whether policy is followed, who is involved & who has a voice can all lead to exposure to MD. Discomfort at decisions made by others & not confronting them. | Not necessarily negative - high levels of job satisfaction, strong group cohesion & an effective work life balance contributed to a reduction in feelings of stress. | Joint decisions making key to effective communication & risk of exposure to MD. | *Not reported.* | *Not reported.* | Importance & benefits of shared decision making. Highlighted the importance of enhancing communication through training & further research, collaborative education, debriefing & team meetings to improve dynamics within the team. |
| Ritchie et al., (2018), Canada | What are the experiences of MD in nurse practitioners in continuing care? What are contributing factors & issues experienced. | 6 nursing practitioners’ in community care & supported living settings/ purposeful sampling. | Mean age 44 yrs*/* All female/  *Ethnicity not reported/ Religion not reported.* | Semi-structured interviews. | Interpretive description. | 1. Patients.  2. Perceptions.  3. Physicians.  4. Palliation.  5. Policies. | Jameton initially defined MD as when “one knows the right thing to do, but institutional constraints make it nearly impossible to pursue the right course of action”. | Patients – provision of good care. Perceptions – tensions that arise between nurses, family members & managers. Physicians – power struggle between doctors & nurses.  Palliation – struggle to meet the multiple dem&s in palliative care. Policies – tensions form between expectations & responsibilities. | Moral residue. | Peer discussion, self-evaluation, teach others about their role, having advanced practice nurses in leadership roles. | *Not reported.* | Small sample of only those in community care. The term MD was interpreted differently by each participant. | Nursing practitioners can play a pivotal role in meeting the dem&s of an ageing population in community settings. Barriers need to be addressed through education & research. |
| Robinson & Stinson, (2016), USA | Determine how emergency care nurses define MD, & examine the experiences of MD, its impact, & identify possible strategies to combat MD. | 9 nurses in 3 different ED’s/ convenience sampling. | *Age not reporter/* 3 men, 6 female/ *Ethnicity not reported/ Religion not reported.* | Structured in-depth interviews. | Phenomenological approach. | 1. There was no face of the family.  2. Asking God for forgiveness. 3. Flipping the switch.  4. It changes who we are. | Jameton described MD as feelings that are painful leading to a psychological imbalance or disequilibrium that occurs when nurses find themselves in situations where they believe they are unable to do the right thing. | Patient advocacy issues, professional behaviour of other health care professionals, internal conflicts with what they perceived to be the right thing to do, & guilt over their own feelings about patients & patient care. The nurses reported specific incidents, rather than environmental factors, as sources of MD for them. Nurses often reflected that they did not have time to address their feelings during a shift & had to move to the next patient without time to recover emotionally. | Family & professional life grossly affected. Nurses felt closed off from others. Feelings of guilt associated with their reactions to stress. | "Something needs to be done" - no specific intervention mentioned, help needs to come from those nurses who work on the shop floor, group debriefs/time for discussions & de-stress, leaders awareness of the situation, education. | Conflict between what they believe is right & decisions made by others, be it other health care providers, patients ‘families, or patients themselves. | Small number of registered nurses in 1 area of the country, may not represent views of all emergency nurses. Nurses participating in the study may have felt the need to respond in a professional way during the interviews to avoid representing themselves negatively. | MD is a frequent & troubling issue in emergency nursing, nurse leaders need to be aware that MD is a real & frequent threat to the well-being of nurses, nurses & nurse leaders need to learn to recognise situations that may cause MD & recognise symptoms of MD in themselves & in their colleagues & should work together to support nurses who are experiencing it. |
| Scott et al., (2023), UK | "seeks to: 1. Explore the sources of moral distress experienced by critical care staff during the Covid-19 pandemic.  2. Identify the meaning that critical care staff attached to the sources of moral distress and any factors which provide support when dealing with such challenges." | 17 nurses and AHPs and doctors in NE of England hospitals/ not reported | nurses mean age of 29.5 years - AHP mean age 39 - doctors mean age 44.3 / 12F 3M/ not reported | semi-structured interviews | constructivist paradigm and naturalistic inquiry / reflexive thematic analysis | "source of moral distress from concerns about personal and family safety related to the infective nature of the virus, a lack of confidence about  Personal Protective Equipment, as well as a high number of death and concerns about care delivery. The consequences for participants and their colleagues were also articulated > 5 themes: Counterintuitive care, Tough days, Not the usual standard, Personal and Family Safety, Consequence of  moral distress" | Moral distress occurs when a health professional experiences a situation which conflicts with the morally accepted norms for care delivery. The concept was first conceptualized in healthcare by Jameton (1984) who described it as a psychological discomfort that occurs when a person is unable to complete what they believe to be an ethically responsible action due to internal or external factors | "presentation of the patients and the counterintuitive nature of the care guidelines... suddenly deteriorated and died or when there was a high number of deaths... they were delivering care that they regarded as sub-standard... availability and quality of Personal Protective Equipment as  well as concerns for the safety of family members" | "psycho/physical manifestations of fatigue (burnt out and many experienced low mood, depression, and flash-back episodes suggesting significant moral injury and other psychiatric  problems.)" | "Leadership integrity  acts as a buffer for moral distress providing staff with reassurance  that colleagues in leadership positions are watching  out for them during times of stress and difficulty" | *n/a* | only one critical care department between the first and second (UK) waves of the pandemic... does not measure the frequency of exposure over a specific time period. In addition, the retrospective nature | From the outset systems of support such as de-brief and group support should be implemented from the outset of any infection outbreak. Such approaches go some way to providing staff with initial support and an opportunity to explore morally distressing situations... the value of leadership not only in determining the approach to patient care but also in supporting colleagues through their words and behaviors. important to counterbalance infection prevention and control with appropriate family access (wearing personal protective equipment) and with care delivery. |
| Silverman et al., (2021), USA | Explore the causes of MD in nurses caring for COVID-19 patients, & identify the strategies used to enhance moral resilience. | 31 nurses caring for COVID-19 patients in acute care departments/ purposeful sampling. | 20-30 yrs (45%), 30-40 yrs (29%), 40> yrs (26%)/ *Gender not reported/ Ethnicity not reported/ Religion not reported.* | Focus groups & in-depth interviews. | Thematic framework, apriori categories; individual, relational, organisational & systematic. | 1. Individual characteristics.  2.External constraints. 3. Institutional constraints. 4. Structures & processes. 5. Systemic institutional policies.  5. Coping strategies to address MD: Individual, relational, institutional. | A conceptual framework that shows the interplay between moral sensitivity, MD, moral resiliency & moral agency, & the ethical climate for enhancing the moral resiliency in order to restore moral agency. | Rapid change of environment, overwhelming number of patients, fear of exposure to virus. Nurses’ voices not heard in plan of care decisions. Personal protective equipment (PPE) policy, unable to help families grieve, watching patients die alone. Scarcity of resources, lack of time to grieve. | Sense of chaos, perceived helplessness, lack of knowledge, fear, constraints on nursing dynamics within revolving teams, nurse-physician interactions, conflicts with family. | Establish an ethical climate in the workplace that ensures the institution reflects its ethical values in decisions & behaviour. Combine moral sensitivity & ethical climate to enhance moral agency. Effective leadership to foster a supportive workplace. | *Not reported.* | Zoom meetings have limited in depth discussions in focus groups. May be limited to only the experiences of the nurses within the hospitals studied - as other hospitals varied by how many surges of COVID patients they had surged. | Fundamentally MI relates to organisational structure. "To enhance the moral climate, organizations need to develop strategies for ensuring non-hierarchical interdisciplinary spaces in which all providers can meet as moral peers to address situations of ambiguity & powerlessness that occur in clinical practice." |
| Smith et al (2023), Canada | To identify types of moral distress among women healthcare providers during the COVID-19 pandemic; to explore how feminist political economy might be integrated into the study of moral distress. | 78 HCW and 10 key informant (management)/ various/ purposive | not reported/ all female/ not reported | interviews and focus groups | framework analysis using broad gender-based lens | moral constraints (staffing shortages, unable to ensure safety due to lack of access to PP, unable to ensure children's education and wellbeing, lack of care infrastructure, union organising, advocating for PPE), conflict (unable to advocate because of distanced decision making, lack of flexibility at work, supporting childcare campaigns), dilemma (covid-19 protocols impact on care, betewen unpaid care responsibilities vs. covid-19 risk, counseling), uncertainty (lack of and constantly changing info, professional pride) | Morley et al.’s definition of moral distress as “the combination of (1) the experience of a moral event, (2) the experience of ‘psychological distress’, and (3) a direct causal relation between (1) and (2).”6 Morely et al. identify multiple types of moral events, arguing this broadening of legitimate events leading to moral distress supports the development of preventive and responsive interventions.7 | moral constraints (staffing shortages, unable to ensure safety due to lack of access to PP, unable to ensure children's education and wellbeing, lack of care infrastructure, union organising, advocating for PPE), conflict (unable to advocate because of distanced decision making, lack of flexibility at work, supporting childcare campaigns), dilemma (covid-19 protocols impact on care, betewen unpaid care responsibilities vs. covid-19 risk, counseling), uncertainty (lack of and constantly changing info, professional pride) | *resistance* |  |  | very little discussion | Instead, it adds to the literature by documenting the lived experiences of a specific group identified as at risk of moral distress and contributing meaningful inquiry into discussions around moral distress |
| St Ledger et al., (2021), UK | Explore the triggers for MD in end-of-life care, & the personal & organisational constraints that influence triggers, & consequence for individuals involved. | 18 physicians specialising in ICU/ purposeful sampling. | 20-70 yrs of age/ 14 males, 4 females/ *Ethnicity not reported/ Religion not reported.* | Interviews. | Thematic analysis. | 1. Key MD triggers.  2. Strategies & consequences. | An extreme emotional response, when constrained by personal limitations or organisational restrictions from doing what their conscience believes is the ‘right thing’. MD is a complex phenomenon caused by personal & institutional constraints. | MD has different triggers according to physician grade. The decision to admit or not to admit. Breach of physician continuity triggered MD. Prolonged patient case involvement can incur a moral burden for trainees risking MD & burnout. Escalating dem&, increasingly complex cases & resource & autonomy constraints challenge moral integrity. | *Not reported.* | *Not reported.* | *Not reported.* | Sampled at one point in time after the end-of-life care experience & only life limiting case histories were used within this study. Study based at a single centre. | MD can influence a trainee's decision to specialise in intensive care medicine & impact the retention of physicians. Rather than singularly promoting personal resilience, multi interventional approaches, addressing individual & systemic factors, including training, induction, mentorship programmes, organisational climate, & culture. |
| Sukhera et al., (2021), Canada | Explore how resident physicians perceive MD in relation to structural stigma, in any faculty managing the clinical care of COVID-19 patients. | 22 junior doctors in mixed facilities manging the clinical care of COVID-19 patients/ convenience sampling. | *Age not reported/* 4 male, 19 female/ *Ethnicity not reported/ Religion not reported.* | In-depth interviews. | Constructivist grounded theory. | 1. How & why residents experienced MD due to structural stigma.  2. How residents sought to reconcile or respond to their MD.  3. Moral residue & the consequences of MD. | A form of MD called structural distress, focusing on the experience of powerlessness, leading physicians to go above & beyond the call of duty, potentially worsening their psychological well-being. | Causes of MD through structural stigma: restrictive visitor policies, limited access to culturally appropriate services, disproportionate impact of the p&emic on individuals needing mental health care. Powerlessness due to structural changes in policies.. | Attempted to reconcile MD though sharing, advocacy, & by doing extra. Doing extra was described as "maladaptive, unsustainable copings strategies with adverse consequences for mental health". Develop a sense of self-blame, helplessness, & compassion fatigue. | Transparency & inclusivity improve sense of powerlessness. Use of advocacy at a patient & policy level to turn conversation from complaining to constructive behaviour. Mentorship schemes. | *Not reported.* | The p&emic meant it was only possible to recruit through social media. The study was limited to Canada only. The changing nature of the p&emic meant that the experiences & opinions of participants changed as time progressed. | Draws attention to the importance of organisational structure causing MD & how these organisations need to put in place Structural components that will prevent MD & its consequences. |
| Thomas et al., (2016), USA | Explore MD among paediatric clinicians within the context of resuscitation experiences, & rather than previous psychological responses, are there any ethical perspectives of MD that can be conceptualised as challenged to integrity. | 25 clinicians & nurses (5 PICU attendings, 5 CC fellows, 5 PICU nurses,  5 PICU advanced practice providers, 5 PICU respiratory therapists) in a PICU unit/ convenience sampling. | *Age not reported*/ 7 male, 18 female/ *Ethnicity not reported/ Religion not reported.* | Semi-structured interviews. | Thematic analysis. | 1. Lack of underst&ing of the ‘Big Picture’.  2. Variable meanings of ‘Resuscitation’.  3. Reflective team leadership.  4. Uncertainty of role responsibilities. | ''When nurses know the right thing to do, but institutional constraints make it nearly impossible to pursue the right course of action”. | When physicians & nurses internalise “big picture” questions. “Could we have prevented this from happening?” “Should we continue going?” & “Are we doing this for the parents or for the child?”_ uncertainty & disagreement among provider teams about patients’ overall condition & prognosis. Variations in the definitions of “resuscitation”. Uncertainty of role responsibilities negatively impacts performance & contributes to morally distressing situations. | Feelings of anguish, fear,  guilt, sleeplessness, anger, sadness, frustration, powerlessness, & loneliness. | Value a clear hierarchy, in which the team leader explicitly takes the responsibility to direct & regulate team dynamics. Clinicians should be encouraged to also discuss & reflect upon the challenges of professional integrity at the bedside. | *Not reported.* | Recall bias is inherent in human nature regardless of timeframe. Participation in the interviews was voluntary & the perspectives of our clinicians may be different from those who chose not to participate. Those who volunteered may have been more reflective on MD & their professional roles, open to sharing their narratives. | *Not reported.* |
| Thorne et al., (2018), Canada | Critically explore the dynamics of experiences of MD in neonatal ICU, what can be learned about MD from professionals working in the setting. | 28 (15 physicians, 6 clinical associates, 5 nurses, 4 respiratory therapists, 3 social workers & 1 pharmacist) in a neonatal ICU/ convenience & snowball sampling. | 30-70 yrs of age/ 8 males, 19, 1 unreported/ *Ethnicity not reported/ Religion not reported.* | Semi-structured interviews. | Interpretive description. | 1. Nature of the problem.  2. Human impact patterns: Responding & reacting. | MD occurs when one knows the right course of action, but institutional or cultural constraints prevent one from pursuing it. | The NICU context that created specific triggers for MD. These included kinds of ethically complex clinical scenarios, & also a set of organisational & relational conditions within the workplace culture in which those complex clinical scenarios were managed. | Managing MD was very much a part of managing the entirety of the emotional residue of work. There are narratives of bursting into tears, nightmares, broken relationships, depression, anxiety, & self-medication. | Coping through avoidance - protective coping, avoiding & blunting. Interactional coping - reframing & rebooting. Existential coping - reflecting & philosophising. | *Not reported.* | Recognised that we will not have captured all possible clinical scenarios or responses to them that might contribute to MD among those who work in such a setting. We also know that each NICU will have its own distinctive culture & working climate. | MD was a prominent & pervasive experiential aspect of work in the NICU. It was also learned that it was difficult to discuss further exploration & evidence building around how best to lead & manage NICU to minimise the consequences of power imbalances. |
| Trachtenberg et al., (2022), USA | Explore experiences of nurses during care of Covid patients in ICU, during the 1st surge of p&emic. Explore how the concept of MD may exp& to account for experiences during p&emic. | 20 (16 nurses & 4 respiratory therapists) in 2 ICU’s at an academic medical centre/ purposeful sampling. | *(2 participants did not give demographic info).*  Mean age 32.8 (SD=8.8)/ 1 male, 17 women/ 16 white, 2 black/ *Religion not reported.* | In-depth semi-structured interviews. | Inductive & deductive approach. | 1. Fear of the unknown (lack of knowledge about the virus & changing protocols).  2. Concerns about infection (spread to selves & family).  3. Perceptions of professional unpreparedness, isolation & alienation (inside & outside hospital).  4. Inescapable stress & distress (no time for rest & recovery). | Jameton. | *Not reported.* | *Not reported.* | *Not reported.* | *Not reported,* | *Not reported* | *Not reported.* |
| Villa et al., (2021), Italy | Describe the experience of MD in all types of healthcare professionals providing daily elderly care within the community & hospitals. | 13 (5 nurses, 3 physicians, 2 nursing assistants, 1 educator, 1 physiotherapist, 1 psychologist) from a hospital (6) & a nursing home (7)/ purposeful sampling. | 18-34 yrs (5), 35-49 yrs (2), 50-69 yrs (6)/ 5 male, 6 female/ All Caucasian/ *Religion not reported* . | Interviews. | Grounded theory. | 1. Talking & listening.  2. Care provider wellbeing.  3. Decision making.  4. Protective factors.  5. Potential solutions - core category of “sharing daily”. | MD is the psychological distress of being in a situation in which a healthcare professional is constrained from acting on what he/she knows to be right. | Scarcity of operators undermine the safety of patients & professional, withdrawal from patient care. Conflicting dem&s from patients & family members, patient opposition to care & assistance. Poor quality of care, such as limited resources. | Consequences attributable to distraction & isolation, both at work & at home. Depression, crying, closure towards relationships, residual effects of distress after work. | Personal characteristics, professional characteristics, support from leaders, psychological support, relational skills. Positive social relationships, education can help to learn how to prevent or manage the consequences. Faith & spirituality. | *Not reported.* | The results are not generalisable, but applicable to similar contexts. | Managers can be involved in activities to improve interprofessional reflections among practitioners. Discussing real cases, including healthcare leaders to promote knowledge of MD starting from degree courses. |
| Walt et al., (2022), USA | Examine how clinicians experience MD when deciding to use Section 35 for patients with substance abuse disorders. | 21 (7 physicians, 6 psychiatrists/psychologists, 4 social workers, 2 interns, 2 physician assistants) in 5 hospitals/ convenience sampling. | *None reported.* | Semi-structured in-depth interviews. | Thematic/ narrative analysis. | 1. Justifying clinical decisions across a spectrum of MD.  2. Prioritizing physical over socioemotional wellbeing in the emergency department.  3. Systemic barriers to utilizing Section 35.  4. Strategies for coping with MD. | The negative feeling that occurs when a clinician is required to pursue a treatment option against their moral judgement due to institutional constraint. | Not expressing the inability to act in accordance with their best clinical judgement. Hesitation towards using Section 35. Remaining emotionally neutral in clinical interactions. Deep discomfort with Section 35 that was exacerbated when clinicians’ had limited alternatives & utilised the policy when they did not believe it was the best clinical option. | *Not reported.* | Team-based approach & using previous experiences to inform their decision-making. An additional strategy clinicians utilised to reduce MD with Section 35, was to reassure themselves they only make this decision as a last resort. | *Not reported.* | Research focused on Section 35 use in urban areas, study did not ask clinicians to self-identify their gender, study did not specifically probe about clinicians’ experiences & perspectives on Section 35 in relation to specific substances. No consequences of MD, study did not ask probing questions about power dynamics among clinicians. | Most clinicians expressed concern & discomfort with the criminal aspects of Section 35, which highlights the importance of restructuring Section 35 such that it does not reflect a criminal proceeding. |
| Weiste et al., (2023) | study the ways in which elderly care practitioners discuss moral distress in their work | 3 unit supervisors and 35 care workers in 3 elderly care units / unclear | *not reported* | focus groups/ in-depth workshop discussions | interaction-oriented / conversational and discursive analysis | MD in relation to client work and teamwork. Three main topics were identified - the power to influence and make decisions, equal treatment of everyone, and collaboration. Within teamwork, MD in relation to oppportunities to influence and low autonomy. Poor collaboration e.g. lack of support from co-workers and interpersonal conflict. Leadership styles that emphasise mistakes as learning opportunities and a joint approach to reducing MD. MD experiences associated with anger, frustration and negotiation of perceptions of right and wrongs of care work. The authors conclude that wider discussions about MD are key in reducing MD within a context of psychological safety, in which sharing of negative expereinces can be done within a supportive team. They also point to the importance of from individual problems to institutional solutions (organisational improvements). | Jameton | Refers to Jameton then talk about more recent qual work that looks at disagreements over clinical decisions, use of coercive methods and lack of consent, as well as organisational factors such as lack of resource, poor staffing and low autonomy. Cites individual factors as age, experience, competence and moral sensitivity. Lack of ethical competence (skills, knowledge and willingness to act according to one's own responsibilities may enhance MD. |  | Institutional solutions, for example providing the space to release emotions and focus on problems (through discussion) to help create organisational solutions to work-related wellbeing. Shift from individual problems to institutional solutions. | *n/a* | Incorrectly talks about small sample size and lack of generalisability. Lack of demographic info on participants. | Emotional outbursts are superceded by challenges at the workplace level in terms of causing MD. Reflecting and sharing the emotional load are important for practitioner wellbeing. Practitioners need opportunities for problem solving and to give imput into organisational level changes. |
