# Supplementary material for: Triggers and factors associated with moral distress and moral injury in health and social care workers: A systematic review of qualitative studies
Source: PLoS One. 2024 Jun 27;19(6):e0303013. doi: 10.1371/journal.pone.0303013 (PMC11210881; doi:10.1371/journal.pone.0303013)
Supplement: Attachment — Submitted filename: PLOS ONE01.09._comments.docx [file pone.0303013.s004.docx]

PLOS ONE

Experiences of moral injury in healthcare workers: A rapid systematic review of qualitative studies

**In general, it is important to provide an overview of the experience of moral distress and moral injury, causes, and the consequences among health care workers especially with the overview of qualitative studies, insights into the experience of health care workers can provide a deeper understanding of the constructs of MI and MD. Nevertheless, the manuscript needs to be fundamentally revised.**

**Title**

- Only MI is addressed in the title. Why is MD not mentioned here? Perhaps it would be better to mention MD as well, since the main studies also use only this terminology?

**Introduction**

- Line 78: Please add a short definition of Compassion Fatigue.
- In the definition of MI the references are completely missing (Line 80-86). Which definitions were used here?

**Aims and objectives**

- The secondary objectives differ from those available online in the review protocol. Please adjust the information.

**Method**

- Why was a Rapid Review conducted when the survey period was June 2021 to February 23?

**inclusion exclusion**

- Again, there is a discrepancy between the manuscript and the review protocol online. Please standardize here accordingly.
- Were social workers included or excluded?
- What are "health facility managers"? Why were they included and management not?

**Risk for bias/ quality assessment**

- The link for the CASP tool is not up to date. Please add a new link.

**Results**

Overall it would be nice if the presentation of results is something more precise and stringent. A brief summary of how many and which studies addressed each of the topics listed below might help. For example, in line 303 you wrote "few". Out of 46, only 3 mentioned MI. This is a result worth mentioning, (which may also have relevance to both the title of the review and the discussion at the end). Also, I would appreciate if individual factors of the dimensions and categories were discussed, as they cannot be exclusively assigned to one category (see below for a detailed note/description).

**Theme 1: Causes and Triggers.**

- A lack of time is classified as a personal trigger. Why is this not classified as an organizational trigger?
- Line 402: Here, the lack of trust is classified as a trigger. In the literature, the experience of betrayal is the cause of the loss of trust (as an experience). Here I would just like to see a separation betrayal as cause for the loss of trust as experience or consequence.

**Experiences**

- Lines 426/427 here it says that people describe their experience of MD and MI in metaphors. Can't that storytelling also be understood in the meaning making context and thus as coping or learning? Perhaps one could at least discuss the point.
- Sometimes the quotes are also not meaningful line 434 " plenty of emotional distress" does not characterize the distress in detail. Perhaps a statement could be picked out here which really characterizes emotional experience.
- If the specific negative emotions were mentioned in only a few of the studies examined, this would also be an interesting result, but this result should be named clearly. Maybe as “non-specific negative”?
- In the quote from line 462/ 463, questions are raised not only about the moral " role" but also about the moral self. This could then be mentioned in the text/graphic as well!

**Consequences**

- Please add references (Lines 491,499)
- Line 511: Here short-term experience and longer-term experience are mixed up. Please have a look at the syndrome perspective of Jinkerson or the conceptualization of Litz et al.
- Learning from previous experiences can also be coping. Probably discuss this issue (lines 522-524)

**Secondary objectives**

**Diversity/cultural differences and moral injury/distress.**

- Here they only looked at triggers for MD and MI. Have differences been found in the experience of MD and MI? Are there differences in emotions or differences in physiological response?

**Major events/disasters (e.g., pandemics, low probability/high impact events) and 574 potential for influencing moral distress and injury experiences.**

- Line 581 please add a reference

**reported coping and treatment strategies and/or interventions.**

- Please add a reference at line 628. Also, from 666-667 it would be interesting to see the articles that have examined this.
- At 653 it is said that ethical training can be useful or not. How do the authors see this, how do they justify this and please also a reference.

**Discussion**

In this section, it would be desirable to discuss the conceptual difficulties of experiencing, consequences, and coping strategy. These become especially evident in the emotional experience, in the social consequences (withdrawal or job abandonment), or in the reinterpretation (once in the experience and once in the learning). It would be good if at least the difficulty of the exclusivity of the categories could be addressed.

- In line 694 I would like to have a reference source again.
- Line 727 here talks about the buffering effect of resilience. Resilience also includes other components that I think could be addressed here.

**Implications for practice**

- Almost the entire section lacks references to the implications that have already been made in the existing studies. Please add them.

**Formal aspects:**

- Please check the whole manuscript for spelling (753).
- Please use consistent spelling (e.g. lines 41,95; 115,120,575,682). Please write out the constructs at the first mention and the abbreviation in brackets (e.g. line 90,170).
- It would be nice if you could unify the headings in the style (line 233, line 243) (348 or 421).
- For the citations in the results section, it would be nice if you also refer the lines of the quotes.

**Formal aspects of tables and graphics**

- Please add a legend to table 2. This could explain the terms ICU and IPA, MD and MI. It would also be nice if the headings of the columns were repeated on each page to improve readability.
