# Supplementary material for: Triggers and factors associated with moral distress and moral injury in health and social care workers: A systematic review of qualitative studies
Source: PLoS One. 2024 Jun 27;19(6):e0303013. doi: 10.1371/journal.pone.0303013 (PMC11210881; doi:10.1371/journal.pone.0303013)
Supplement: Attachment — Submitted filename: response to reviewers.docx [file pone.0303013.s005.docx]

Dear Editors,

Thank you for giving us the opportunity to submit a revised draft of the manuscript ‘’Experiences of moral injury in healthcare workers: A rapid systematic review of qualitative studies’’ that is now titled **“Triggers and factors associated with moral distress and moral injury in health and social care workers: a systematic review of qualitative studies”** for publication in PlosOne. We appreciate the time and effort that you and reviewers have taken to provide us this feedback and are grateful to you for insightful comments on and valuable improvements which we believe have been made to our paper. We have incorporated most of the suggestions made by the reviewers. Please see below, in blue, for a point-by-point response to the reviewers’ comments and concerns. All page numbers refer to the revised manuscript without changes.

Reviewer’s comments to the authors:

**Editor comments:**

1. Please ensure that your manuscript meets PLOS ONE's style requirements, including those for file naming.
2. We noted in your submission details that a portion of your manuscript may have been presented or published elsewhere. "This work is a review, as such the data is compiled from the reviewed papers and quotes are published in these respective papers, cited appropriately where used." Please clarify whether this [conference proceeding or publication] was peer-reviewed and formally published. If this work was previously peer-reviewed and published, in the cover letter please provide the reason that this work does not constitute dual publication and should be included in the current manuscript.

We thank the editor for this comment, and we want to clarify that, this being a review article, the content of the manuscript (beyond the quotes published in relevant papers) is not published anywhere else in any format.

3. Please upload a new copy of Figures 1 and 2 as the detail is not clear.

The figures were reformatted, and we changed figure 2 and added figure 3 as a result of the work on the content based on reviewers’ comments. The figures are now formatted as required.

**Reviewer 1:**

In general, it is important to provide an overview of the experience of moral distress and moral injury, causes, and the consequences among health care workers especially with the overview of qualitative studies, insights into the experience of health care workers can provide a deeper understanding of the constructs of MI and MD. Nevertheless, the manuscript needs to be fundamentally revised. For details see attachment

We thank the reviewer for the thorough review of the paper and for the comment on the importance of the topic and its relevance in current times when accounts of professionals’ distress are many. We agree the revision of the manuscript was necessary to improve the impact it will have on the body of literature on moral distress. We revised it thoroughly and made further improvements, triggered by the reviewers’ comments.

**Title**

- Only MI is addressed in the title. Why is MD not mentioned here? Perhaps it would be better to mention MD as well, since the main studies also use only this terminology?

The title has been revised and now mentions the modified focus. Moral distress is now added.

**Introduction**

- Line 78: Please add a short definition of Compassion Fatigue. We thank the reviewer for this comment, this was addressed, and definition included.
- In the definition of MI the references are completely missing (Line 80-86). Which definitions were used here? We thank the reviewer for this comment, this was addressed, and references included.

**Aims and objectives**

- The secondary objectives differ from those available online in the review protocol. Please adjust the information. We thank the reviewer for this comment, this was addressed, and a new protocol on Prospero has been created. It is currently under review as of submission, and a new link will be provided prior to publication.

**Method**

- Why was a Rapid Review conducted when the survey period was June 2021 to February 23? We thank the reviewer for this comment. As mentioned, we changed this from rapid to a systematic review and the methods section changed accordingly.

**inclusion exclusion**

- Again, there is a discrepancy between the manuscript and the review protocol online. Please standardize here accordingly. We thank the reviewer for this and the comments below. This was addressed, and new details were added in the new revised protocol.
- Were social workers included or excluded? Social workers were included, and this is mentioned in the text.
- What are "health facility managers"? Why were they included and management not? We agree that this was misleading, and we changed the eligibility criteria accordingly.

**Risk for bias/ quality assessment**

- The link for the CASP tool is not up to date. Please add a new link. We updated the link as suggested.

**Results**

Overall it would be nice if the presentation of results is something more precise and stringent. A brief summary of how many and which studies addressed each of the topics listed below might help. For example, in line 303 you wrote "few". Out of 46, only 3 mentioned MI. This is a result worth mentioning, (which may also have relevance to both the title of the review and the discussion at the end). Also, I would appreciate if individual factors of the dimensions and categories were discussed, as they cannot be exclusively assigned to one category (see below for a detailed note/description).

We thank the reviewer for these suggestions. Authors met and discussed how this manuscript could be improved in terms of narrative and content. We realised the most important addition to the body of literature our review is introducing is the thorough investigation of cause and triggers of moral distress. We re-arranged the results section and, as suggested, we discussed the categories and sub-categories. The issue of assigning elements to one category was also discussed and reported in the manuscript. A table with reference to how many files mentioned the sub-categories was added.

**Theme 1: Causes and Triggers.**

- A lack of time is classified as a personal trigger. Why is this not classified as an organizational trigger?

The issue of assigning elements to one category is real and we had conversations around those elements we could not assign easily. We changed the themes tree to a figure that indicates the overlapping of sub-categories. [Figure 3]

- Line 402: Here, the lack of trust is classified as a trigger. In the literature, the experience of betrayal is the cause of the loss of trust (as an experience). Here I would just like to see a separation betrayal as cause for the loss of trust as experience or consequence.

We consider the element of trust and its link with betrayal. We hope the narrative now reflects this link.

**Experiences**

- Lines 426/427 here it says that people describe their experience of MD and MI in metaphors. Can't that storytelling also be understood in the meaning making context and thus as coping or learning? Perhaps one could at least discuss the point.
- Sometimes the quotes are also not meaningful line 434 " plenty of emotional distress" does not characterize the distress in detail. Perhaps a statement could be picked out here which really characterizes emotional experience.
- If the specific negative emotions were mentioned in only a few of the studies examined, this would also be an interesting result, but this result should be named clearly. Maybe as “non-specific negative”?
- In the quote from line 462/ 463, questions are raised not only about the moral " role" but also about the moral self. This could then be mentioned in the text/graphic as well!

We decided to change the focus of the review and report the cause and triggers of moral distress/injury. We noticed, from reviewers' comments, that we had too much content and we could not address properly the numerous concepts that ‘’experience’’ and ‘’consequences’’ constructs entail. We decided to restrict the report and look to those elements that did not have the same amount of literature published on. The moral self was also mentioned and discussed briefly.

**Consequences**

- Please add references (Lines 491,499)
- Line 511: Here short-term experience and longer-term experience are mixed up. Please have a look at the syndrome perspective of Jinkerson or the conceptualization of Litz et al.
- Learning from previous experiences can also be coping. Probably discuss this issue (lines 522-524)

Similarly to the above comment, we refocused the review report to address a gap in literature about a review of potential causes and triggers.

**Secondary objectives**

**Diversity/cultural differences and moral injury/distress.**

- Here they only looked at triggers for MD and MI. Have differences been found in the experience of MD and MI? Are there differences in emotions or differences in physiological response?

We replied to this query adding a comment in the results section that assumptions were not possible since we could not retrieve data linking participants characteristics and MD/I.

**Major events/disasters (e.g., pandemics, low probability/high impact events) and 574 potential for influencing moral distress and injury experiences.**

- Line 581 please add a reference

Thanks for this suggestion, we added a reference.

**reported coping and treatment strategies and/or interventions.**

- Please add a reference at line 628. Also, from 666-667 it would be interesting to see the articles that have examined this.

Thanks for this suggestion, we added references as needed.

- At 653 it is said that ethical training can be useful or not. How do the authors see this, how do they justify this and please also a reference.

The coping strategies section has been reworked according to the three main categories of individual, social, and organisational domain to reflect the causes and triggers section. Therefore, some concepts or conflicting messages are not present anymore.

**Discussion**

In this section, it would be desirable to discuss the conceptual difficulties of experiencing, consequences, and coping strategy. These become especially evident in the emotional experience, in the social consequences (withdrawal or job abandonment), or in the reinterpretation (once in the experience and once in the learning). It would be good if at least the difficulty of the exclusivity of the categories could be addressed.

- In line 694 I would like to have a reference source again.
- Line 727 here talks about the buffering effect of resilience. Resilience also includes other components that I think could be addressed here.

This section has now been reworked according to the analysis reported in the results section.

**Implications for practice**

- Almost the entire section lacks references to the implications that have already been made in the existing studies. Please add them.

We decided to go beyond the findings and suggestions of reviewed papers and focus on participants’ quotes. The new analysis enabled us to reframe the causes and triggers, and go deeper in finding aggregate themes. We framed the implications for practice on those themes.

**Formal aspects:**

These comments were considered; we were not able to retrieve the lines of the quotes from original papers.

- Please check the whole manuscript for spelling (753).
- Please use consistent spelling (e.g. lines 41,95; 115,120,575,682). Please write out the constructs at the first mention and the abbreviation in brackets (e.g. line 90,170).
- It would be nice if you could unify the headings in the style (line 233, line 243) (348 or 421).
- For the citations in the results section, it would be nice if you also refer the lines of the quotes.

**Formal aspects of tables and graphics**

These comments have been taken into consideration; it is not possible to know how the table will be split into the final format, therefore we will consider this when editing personnel will inform us about the issue of headings.

- Please add a legend to table 2. This could explain the terms ICU and IPA, MD and MI. It would also be nice if the headings of the columns were repeated on each page to improve readability.

**Reviewer 2:**

Thank you for allowing me to read this manuscript, PONE-D-23-19884. Experiences of moral injury in healthcare workers: A rapid systematic review of qualitative studies

1. The study presents the results of the original research.

Yes, the study is a rapid review of qualitative studies.

As a reader, the introduction/background leads me to the purpose. This rapid systematic literature study examined Experiences of moral injury in healthcare workers. A literature review's strength is adding knowledge to the current database of knowledge.

We thank the reviewer for these comments.

2. Results reported have not been published elsewhere.

Not the result of this review.

3. Experiments, statistics, and other analyses are performed to a high technical standard and are described insufficient detail.

The method section is well described and follows recommended steps. The Prospero should be added to the list of references, as well as the CASP.

Figures, tables and Flowchart are precise. Quality assessment, according to CASP, is clear.

We thank the reviewer for this comment. We added the Prospero and CASP links to the references list.

The analysis: Please clarify what kind of analysis you have performed. Thematic analysis, according to Thomas and Harden? The references state the synthesis should be performed via three stages which overlap to some degree: the free line-by-line coding of the findings of primary studies, the organisation of these 'free codes' into related areas to construct 'descriptive' themes, and the development of 'analytical' themes. Another reference you refer to is Sadlowski and Leeman, and they state consequences, etc, are, at this point, simply words used to encompass segments of data researchers saw (i.e., coded) as belonging together. Although a single word such as consequence “may name an idea, [that word] does not operate as an idea until it is put into a sentence or assertion. An idea/theme needs a subject and a predicate before you can use it as a basis of understanding. “

We thank the reviewer for this comment. It helped us revisit the section of methods and we hope it is now clearer.

Result: Figure 2 needs to be more readable, with too much text and too small figures. Definition of moral distress and moral injury should be placed in the introduction- that will guide analysis later on. Presenting the participant's view?? Does this connect to the primary aim?

We thank the reviewer for these comments. We now changed figure 2 (the alternative one is now figure 3) and erased the sections on definitions because they were not linked to the primary aim.

Interesting findings/themes. However, are they themes? Causes and Triggers, Experiences. Consequences. According to the reference you use—see above. Are they not simplified to answer the aim of identifying reasons for and factors that influence moral injury and distress in HCWs? According to Thomas and Harden, you are supposed to go below the text to be able to describe the theme. Reading the quotations, there is more than one word labelling the theme, saying nothing.

Cause and triggers are also about experiences. The second aim moves forward better, but the themes are left out, and the research questions are used as theme labels.

What kind of analysis and synthesis is this?

The analytical themes are good. So please connect them with the descriptive themes. Now, there is a gulf between them.

Discussion. There is much repetition of the result- referring to the included studies. I am not surprised that your result agrees with other studies and media. So, what new understanding or knowledge did you bring forth?

We thank the reviewer for these comments. We reviewed the sections named above and focus on the revised primary aim. We made the analysis process and themes clearer, and defined the sub-categories the themes derived from. We separated the quotes for causes and triggers, adding a table, to make the reading easier and clearer.

4. Conclusions are presented in an appropriate fashion and are supported by the data.

The author states that this review has highlighted how healthcare workers experience moral distress and injury in their own words. Details of the roles of individual, social and organisational factors in causing distress and moral injury, the experience and the associated emotions, the consequences of experiencing moral conflict and how HCWs cope have been reported. This is just another way to present the objectives. The research questions used were entirely quantitative, and this conclusion suggests that this review has highlighted that more research is required to examine the effects of culture and diversity on the experience of moral injury and distress. (once more a quantitative perspective). The conclusion should have highlighted the analytical themes.

We thank the reviewer for these comments.

We appreciated the suggestion regarding recommendations and further comments the reviewer provided to the editor. We confirm this review has a qualitative nature and we now think this is clearer. We reworked the conclusion based on suggested improvements.

5. The article is presented in an intelligible fashion and is written in standard English. Yes
6. The research meets all applicable standards for the ethics of experimentation and research integrity. Yes
7. The article adheres to appropriate reporting guidelines and community standards for data availability. Style and format OK Clarity -Yes
8. Other comments: Advice for improvement of the manuscript?

There is a need to clarify the method section and present the result.(then discussion etc will also be improved).

There are 92 references used in this paper, and out of these, 20 are old, ten years or more; 19 are used in the introduction and out of these, 6 are about 10 years old. I cannot find the year of publication in the list for references 5,78 and 87.

We addressed these issues, thank you. We excluded papers that were older than 10years since the first search of databases. We also included a figure to show publication year of included papers.

This manuscript would contribute to our knowledge base if the data were analysed and handled according to the references used. I am looking forward to see an amended manuscript

We thank the reviewer for this comment. We are confident the manuscript is improved, and it contributes to knowledge on moral distress and injury.
