# Supplementary material for: Triggers and factors associated with moral distress and moral injury in health and social care workers: A systematic review of qualitative studies
Source: PLoS One. 2024 Jun 27;19(6):e0303013. doi: 10.1371/journal.pone.0303013 (PMC11210881; doi:10.1371/journal.pone.0303013)
Supplement: Attachment — Submitted filename: response to reviewers _EBeadle_April24.docx [file pone.0303013.s006.docx]

Dear Editors,

Thank you for giving us the opportunity to submit a revised draft of the manuscript **“Triggers and factors associated with moral distress and moral injury in health and social care workers: a systematic review of qualitative studies”** for publication in PlosOne. We appreciate the time and effort that you and reviewers have taken to provide us with this feedback and are grateful for the comments. We have incorporated most of the suggestions made by the reviewers. Please see below, in blue, a point-by-point response to the reviewers’ comments and concerns regarding the meaning of some comments.

**Reviewer’s comments to the authors:**

**Journal Requirements:**

Please review your reference list to ensure that it is complete and correct. If you have cited papers that have been retracted, please include the rationale for doing so in the manuscript text, or remove these references and replace them with relevant current references. Any changes to the reference list should be mentioned in the rebuttal letter that accompanies your revised manuscript. If you need to cite a retracted article, indicate the article’s retracted status in the References list and also include a citation and full reference for the retraction notice.

We reviewed the references list to ensure that any retracted references are removed and replaced.

**Reviewer 2 comments:**

4. Have the authors made all data underlying the findings in their manuscript fully available?

The PLOS Data policy requires authors to make all data underlying the findings described in their manuscript fully available without restriction, with rare exception (please refer to the Data Availability Statement in the manuscript PDF file). The data should be provided as part of the manuscript or its supporting information, or deposited to a public repository. For example, in addition to summary statistics, the data points behind means, medians and variance measures should be available. If there are restrictions on publicly sharing data—e.g. participant privacy or use of data from a third party—those must be specified.

Reviewer #2: No

All data we are able to provide are uploaded in the form of supplementary data tables and selected quotes. This is a review, so it would not be possible or appropriate to include the entire contents of the included papers before asking permission to authors with great delay in the submission of this paper to this journal. All this information is available from the selected and referenced original papers.

To support transparency, we have added the updated protocol to the supplementary materials to ensure the most up-to-date version is available to all readers.

Reviewer #2: Most reviewer comments have been considered, and the paper has been reworked and rewritten. So, the paper is amended, but some issues still need to be solved.

Table 3 is clear, but what does the reference figure highlight? If 37 articles are used to get the quotations, what are the references?—171?

The word ‘’reference’’ in table 3 has been given further explanation in the text.

Another area for improvement is how I, as a reader, will judge or understand this review's quality.

There are no or limited references presenting the different articles included in the result, and there are no figures about how many articles built the domains- Table 3 gives some numbers- but there could be repeated articles. Since we do not know how many of the included 51 articles/reports used in this review are used/presented in every domain.

The articles numbers in table 3 do show how many of the 51 articles have made reference to each domain. In some areas, we had not cited all papers (e.g. for categories and coping strategies) because we reported a summary of findings, but we now have ratified this, more references have been added.

In the secondary objective/result, you present some more references—Fifteen articles (39,47–49,52,58,63,64,66,67,72,78–80,83) specifically explored the role of….Nevertheless, mostly, there is no information, and the text presented does not show any evidence that it is coming from the articles when just presenting a few quotations- for example, 4 quotations from 51 articles.

Table 4 shows an example of quotations for each domain. We have changed the Table description to make this clear and added more citations to individual studies in the description of themes and categories. We are not sure that presenting citations from all 51 articles will make the narrative easier for readers, and it is a secondary objective. We summarized the information deriving from quotes to make the content accessible and in line with other qualitative reviews.

The text is supposed to be the analysis from the articles/quotations—the text extraction.

It is; we focused on a description of the themes, since using quotes from 51 articles may not be efficient or easy to read or follow. In the previous draft we inserted the quotes within the text, and it was difficult to follow and understand the main points we were trying to portray. We looked at published literature and decided to insert a table with selected quotes as the best approach we have seen so far.

Forty-three studies explored coping strategies and positive action; please add the references.

These are now listed and the number corrected.

The discussion, well, there is a result presentation- those references presenting the result should be in the result section, and you are supposed to discuss your findings from your review against articles/references from your background or new ones.

We referred to the results in the discussion as thought to be good practice, to link the narrative from one section to another. We introduced some new reference to make clear to the reader that we didn’t infer information pertaining the construct of moral distress from the papers we reviewed, but we focused our analysis of quotations (as explained in methods) to describe triggers and factors. In the introduction we ‘’introduced’’ the topic from a psychological/philosophical point of view drawing from experts’ opinions, then we went beyond that, understanding the triggers and factors producing moral distress in workers to offer practical insights.

There are 23 references from your review result in the discussion- so were only 23 out of 51 articles used to build the result? 3 references from the introduction are found in the discussion and 5 "new" ones.

We understand the listing of 23 references might be confusing, we erased it and made clear that all 51 articles were used in the review, and all are cited. We have clarified this in a few places to make more accessible how this was achieved and where each was used. New references were used when necessary to suggest the reader of potential evidence-based consequences on quality of care and potential solutions that have been found to be effective.

Still, there needs to be some clarification: in the abstract, you state that 51 articles/reports were included in the review- references 39-90 are those articles. However, are 91-93 included in the result as well?

We have checked these references specifically and have ensured that they are correctly cited where relevant. 91 refers to a book used for definitions, this is only cited where this is relevant. 92 is included and cited appropriately now. 93 was mistakenly left in but has now been removed.

In the implications, there are references from the introduction, the result, and a new one. So, what are your implications from the result?

The discussion starts with reference to the construct of moral distress and injury, linking the narrative to the introduction where the concept of moral distress is discussed and adding the definition of moral reasoning (new reference) to provide deeper understanding of different aspects of the problem. We then move to the description of the triggers and factors of distress as emerging from the results, therefore a comparison between the introduction references and the results findings is limited for obvious reasons. The implications from the results are clearly stated in the specific sections.

Limitations of the included articles—the quality of those articles should be judged during the appraisal phase.

This was done and is detailed in the method, results and in table 1. In line with recommendations for structuring of systematic review articles (Page, M.J., McKenzie, J.E., Bossuyt, P.M. et al. The PRISMA 2020 statement: an updated guideline for reporting systematic reviews. *Syst Rev* 10, 89 (2021). <https://doi.org/10.1186/s13643-021-01626-4>). An additional summary of the key issues is highlighted here to help readers know where there is need for improvement in this body of research.

The conclusion is that this is out of the review. There are several aspects I cannot recall from the review results.

Similarly to other points made by this reviewer, the authors struggled to understand the meaning of this comment (is referring to the conclusion of the limitations named above – if so, such conclusion was not made by us) We have inferred that the reviewer thinks some of the conclusions do not accurately reflect, or go beyond what was presented in the results We have addressed this, to better explain and highlight the link between our results and the conclusion.

Still, some years are missing in reference 5—it should be 2021, and reference 102 should be 2022.

We have corrected this.

As I said before, this manuscript would contribute to our knowledge base if the data were analysed and handled according to the references.

A number of appropriate methodology papers have been cited and the process of analysis was followed when compiling this qualitative review and, in our view, thoroughly explained. Having looked at other published PloS qualitative reviews (e.g. Iyahen EO, Omoruyi OO, Rowa-Dewar N, Dobbie F (2023). Exploring the barriers and facilitators to the uptake of smoking cessation services for people in treatment or recovery from problematic drug or alcohol use: A qualitative systematic review. PLoS ONE 18(7): e0288409.), we are confident that our paper provides a similar degree of detail and insight to the processes followed.
